# Post-fire movements of Pacific marten (*Martes caurina*) depend on the severity of landscape change

**DOI:** 10.1186/s40462-021-00286-2

**Published:** 2021-10-09

**Authors:** Logan A. Volkmann, Karen E. Hodges

**Affiliations:** Department of Biology, University of British Columbia Okanagan, Science Building, 1177 Research Road, Kelowna, BC V1V1V7 Canada

**Keywords:** Boreal forest, Carnivores, Fire ecology, Habitat use, Home ranges, Landscape management, Montane forest, Movement ecology, Salvage logging, Wildfire

## Abstract

**Background:**

Wildfires and forestry activities such as post-fire salvage logging are altering North American forests on a massive scale. Habitat change and fragmentation on forested landscapes may threaten forest specialists, such as Pacific marten (*Martes caurina*), that require closed, connected, and highly structured habitats. Although marten use burned landscapes, it is unclear how these animals respond to differing burn severities, or how well they tolerate additional landscape change from salvage logging.

**Methods:**

We used snow tracking and GPS collars to examine marten movements in three large burns in north-central Washington, USA (burned in 2006) and central British Columbia, Canada (burned in 2010 and 2017). We also assessed marten habitat use in relation to areas salvage-logged in the 2010 burn. We evaluated marten path characteristics in relation to post-fire habitat quality, including shifts in behaviour when crossing severely-disturbed habitats. Using GPS locations, we investigated marten home range characteristics and habitat selection in relation to forest cover, burn severity, and salvage logging.

**Results:**

Marten in the 2006 burn shifted from random to directed movement in areas burned at high severity; in BC, they chose highly straight paths when crossing salvage-blocks and meadows. Collared marten structured their home ranges around forest cover and burn severity, avoiding sparsely-covered habitats and selecting areas burned at low severity. Marten selected areas farther from roads in both Washington and BC, selected areas closer to water in the 2006 burn, and strongly avoided salvage-logged areas of the 2010 burn. Marten home ranges overlapped extensively, including two males tracked concurrently in the 2010 burn.

**Conclusions:**

Areas burned at low severity provide critical habitat for marten post-fire. Encouragingly, our results indicate that both male and female marten can maintain home ranges in large burns and use a wide range of post-fire conditions. However, salvage-logged areas are not suitable for marten and may represent significant barriers to foraging and dispersal.

**Supplementary Information:**

The online version contains supplementary material available at 10.1186/s40462-021-00286-2.

## Background

Landscape change is a global and pervasive threat to forest ecosystems [1]. In western North America, disturbances from timber harvest, insect epidemics, and wildfire have caused substantial changes to boreal, sub-boreal, and montane conifer forests (hereafter, “western forests”) over the past 50 years [2, 3]. Large (> 10,000 ha), stand-replacing wildfires are a primary driver of succession in western forests, but such fires were historically rare, typically recurring on timescales of > 100 years [4, 5]. With climate change, these extreme fire events have become increasingly common [6–8], and are likely to play a pivotal role in reshaping North American forest communities [9–11].

Post-fire, the pattern and severity of landscape change matter to wildlife [12–14]. Although fire is a major, beneficial driver of succession in many forests, some forest-associated birds [15], amphibians [16], and mammals [17] respond negatively to post-fire changes in habitat structure. Wildfires change forests by opening the canopy and reducing the structural complexity from trees, snags, coarse woody debris (hereafter, “deadfall”), and understory vegetation [4, 18]. For species that specialize in late-seral (mature and old-growth) habitats, post-fire landscapes may contain fewer suitable habitats. The loss of large trees, for example, reduces the availability of sites for foraging [19], shelter [20], and reproduction [21] within the first few years post-fire. The continued decay and loss of snags post-fire may negatively impact cavity-nesting species over longer timescales [22–24]. However, large wildfires leave a heterogeneous footprint in forests as some areas burn more intensely than others [25]. Some residual habitats in the form of surviving trees, snags, and deadfall persist for decades post-fire, offering prospects for wildlife persistence and recolonization [26–28].

In addition to this complexity, management activities on burned landscapes may further alter the quality of residual habitats. Post-fire salvage logging—the harvest of dead or fire-damaged trees—is a widespread secondary disturbance in fire-prone regions such as British Columbia (BC), Canada [29]. Like wildfire, salvage logging removes forest structure to varying degrees, creating a patchwork of highly-disturbed and residual habitats. However, salvage logging entails a more intensive removal of trees, snags, and deadfall, leaving fundamentally different conditions for wildlife [30, 31]. Critically, it is unclear how many species respond to salvage logging compared to wildfire and conventional timber harvest, despite the increasing prevalence of salvage logging in western forests [32, 33].

Forest specialists such as Canada lynx (*Lynx canadensis*) [34], fishers (*Pekania pennanti*) [35], and marten (*Martes americana* and *M. caurina*) [36] are highly sensitive to landscape change from wildfire and timber harvest. These animals disperse large distances and occupy large territories, meaning that the size, shape, and connectivity of residual habitats shape their behaviour post-disturbance. Narrow forest openings are easier to cross than wide ones, and large patches of residual habitat offer more potential resources than small ones. Thus, differing patterns of burn severity and post-fire salvage logging may affect which parts of the landscape support resident animals [37], serve as travel corridors [38], or entirely exclude certain species [39]. Substantial landscape change post-fire may create ecological traps, where the presence of residual forest structure draws animals into low-quality habitat [40, 41], or perceptual traps, where animals fail to recognize residual high-quality habitat [42].

Marten in particular respond to an array of habitat features across a range of spatial scales, from individual den trees to regenerating cut-blocks [43, 44]. These animals are closely associated with mature and old-growth habitats in western forests, and serve as indicators of ecosystem health in both Canada and the United States [45, 46]. Marten can persist on landscapes altered by timber harvest, using residual forest as “stepping stones” to cross low-quality habitats [47, 48]. They can also use landscapes substantially altered by fire [49–52]. However, habitat features important to marten post-fire have not been clearly identified [53].

On unburned landscapes, marten select home ranges that contain a high degree of structural complexity [43, 44]. Trees, snags, deadfall, and understory vegetation provide critical resources for resting [54], denning [55], avoiding predators [56], and hunting small mammal prey [57]. Within young burns (< 10 years post-fire), marten use sites with abundant deadfall [50] and residual trees [49, 58], suggesting that these animals target areas of low burn severity that are more similar to intact forests. Areas more severely disturbed by fire offer less structural complexity and fewer prey such as southern red-backed voles (*Myodes gapperi*) [59], red squirrels (*Tamiasciurus hudsonicus*) [60], and snowshoe hares (*Lepus americanus*) [61]; the same is true of salvage-logged areas [39, 62, 63]. It is still unclear how marten incorporate this heterogeneity into a home range, or how readily they use low-quality post-fire habitats to reach high-quality ones.

We studied marten movements over three winters in north-central Washington, USA, and central British Columbia, where record-setting wildfires have caused substantial landscape changes over the past 15 years (2005–2020). Our objective was to determine how marten respond to landscape heterogeneity post-fire, by (1) characterizing their movements and home ranges in burned areas, and (2) assessing their tolerance of post-fire salvage logging. We hypothesized that marten would respond to residual forest structure within burns, focusing their activity in areas with remnant trees. Given marten avoidance of open habitats, we expected resident animals to avoid both severely-burned and salvage-logged areas, with stronger avoidance of salvage-blocks than residual stands of snags.

## Methods

### Study areas

We examined marten populations on two post-fire landscapes in Washington (48.790°N, − 119.953°W) and British Columbia (52.071°N, − 122.436°W) that were separated by approximately 400 km. Hereafter we use “Washington” and “BC” to refer to the whole landscape in each region. Our study areas have high-severity fire regimes typical of western sub-boreal and montane forests, with large, stand-replacing fires recurring every 100–300 years on average [4, 5].

**Fig. 1:**
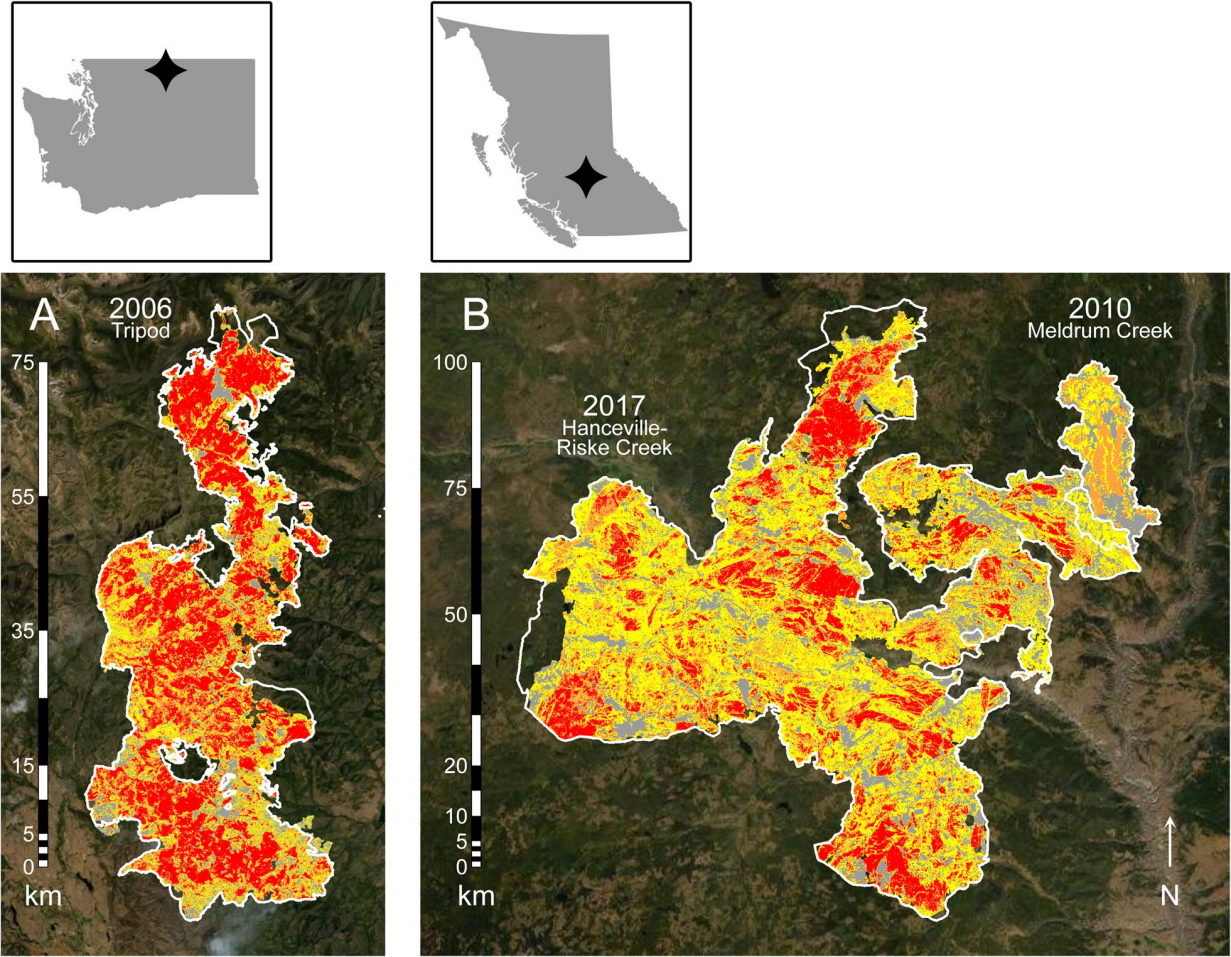
Post-fire landscapes examined in this study: A) the 2006 Tripod Complex wildfire in north-central Washington, USA; B) the 2010 Meldrum Creek and 2017 Hanceville-Riske Creek wildfires in central British Columbia, Canada. White borders denote burn perimeters and map colours are burn severities derived from the differenced Normalized Burn Ratio (2006 and 2010 burns) and Relativized Burn Ratio (2017 burn): gray = unchanged, yellow = low, orange = moderate, and red = high

The 2006 burn, resulting from the 70,575 ha Tripod Complex wildfire, is situated within the Okanogan-Wenatchee National Forest in north-central Washington (Fig. 1; see Additional file 1: Table S1). This landscape has rugged topography and a gradient of forest types typical of the northeastern Cascade Range. Prior to burning, much of the study area was mature, highland conifer forest composed of lodgepole pine (*Pinus contorta*), Engelmann spruce (*Picea engelmannii*), and subalpine fir (*Abies lasiocarpa*), with scattered stands of whitebark pine (*Pinus albicaulis*) and alpine larch (*Larix lyallii*). The regenerating landscape is dominated by snags, deadfall, and an understory of lodgepole pine and willows (*Salix* spp.). At unburned sites, Douglas fir (*Pseudotsuga menziesii*), ponderosa pine (*Pinus ponderosa*), and quaking aspen (*Populus tremuloides*) intergrade at lower elevations and on south-facing slopes; black cottonwood (*Populus trichocarpa*) and bigleaf maple (*Acer macrophyllum*) occur in drainages. Forests transition to alpine parkland above ~ 2100 m, and to shrub-steppe communities below ~ 1000 m [34]. Our study focused on elevations of 1500–2000 m.

The Okanogan-Wenatchee National Forest is not actively managed for timber harvest, with no logging since 1995. A single north–south access road (NF-39), groomed for snowmobile traffic in winter, runs through the center of the study area. Aside from a ~ 10 m road buffer cleared of hazard trees, the 2006 burn has seen little human disturbance post-fire. State forests immediately east of the burn contain mature stands and cut-blocks of various ages, but were not sampled in this study.

The 2010 burn, resulting from the 15,553 ha Meldrum Creek wildfire, and the adjacent 2017 burn, resulting from the 239,340 ha Hanceville-Riske Creek wildfire, are situated on multi-use (Crown) land in central British Columbia, including portions of the Chilcotin Military Reserve (Fig. 1; see Additional file 1: Table S1). This landscape has gently rolling topography dotted with small lakes, and elevations of 900–1000 m. Forests in the area fall under the Interior Douglas-fir and Sub-Boreal Pine-Spruce biogeoclimatic zones [64]; prior to burning, the 2010 and 2017 burns contained mostly mature Douglas fir and lodgepole pine, with smaller amounts of Engelmann spruce, white spruce (*Picea glauca*), and quaking aspen. The regenerating landscape is a mosaic of cut-blocks containing patchy regrowth of lodgepole pine, aspen, and willows, interspersed with islands and stringers of residual trees and snags. Large, natural meadows occur in the southern part of the study area. Approximately 15% of the 2010 burn re-burned in 2017.

The BC study area is actively managed for timber harvest and contains an extensive road network. Virtually all stands of fire-killed trees in the 2010 burn, corresponding to areas that burned at moderate to high severity, were salvage-logged between 2010 and 2012. This activity created a series of salvage-blocks covering ~ 6000 ha and running north–south through the study area. Some selective harvest also occurred in areas that burned at lower severity due to post-fire tree death from mountain pine beetles. Salvage logging at a comparable intensity has taken place in the 2017 burn, beginning in winter 2017–2018.

### Field methods

We examined conditions 10–13 years post-fire for the 2006 burn, 6–9 years post-fire for the 2010 burn, and 0–2 years post-fire for the 2017 burn. Salvage-logged areas of the 2010 burn were 4–9 years old at the time of our study; we did not sample salvage-logged areas of the 2017 burn.

From December to March of 2016–2017, 2017–2018, and 2018–2019, we located marten trails along snowmobile routes in both study areas as part of a larger survey effort for carnivores on post-fire landscapes [65]. Suitable trails ranged from ~ 24 h to several days old depending on snow quality. For each trail, working backwards from the marten’s direction of travel, we recorded the movement path as a series of straight-line 5 m segments (“steps”) marked with pin flags. We georeferenced the trail at its endpoints and at every sixth step (30 m), and measured canopy closure at each of these sites with a spherical densiometer, taking the average of four readings. We prioritized back-tracking in areas > 1 km from previously sampled trails to maximize the number of marten in our dataset and to cover as many potential habitats as possible.

Concurrently with snow tracking, we live-trapped marten using single-door, wire mesh live-traps lined with straw and covered with an open-bottom plywood box and fir branches [66]. We targeted low-severity areas of each burn that frequently had marten tracks, spacing trap sites < 1 km apart and > 50 m from roads. We baited traps with chicken or beaver meat and commercial scent lure, and checked for captures every 24 h. We did not operate traps in ambient temperatures below − 20 °C.

Captured marten were transferred to a vinyl and wire mesh handling cone fitted over the front of the trap [67], and then immobilized via mask induction with isoflurane at an initial concentration of 3% in 1 L/min oxygen using a tabletop vaporizer, a type E portable oxygen cylinder, and a Bain non-rebreathing circuit.

After induction, we moved the marten to a tent containing a heating pad and battery-powered space heater. Handling lasted approximately 20 min at an isoflurane concentration to 1–2% to maintain the desired level of anesthesia; we monitored respiration and rectal temperature throughout. We determined the marten’s sex and body length, and estimated age via visual inspection of tooth wear. Finally, we attached ear tags (Monel #1) in each ear and fitted adult marten with a store-on-board GPS collar / VHF transmitter (Advanced Telemetry Systems Model G10) weighing approximately 30 g. We programmed collars to attempt a satellite fix every 15–20 min for deployments in winter (December–March), or 90 min for deployments until the following winter. The VHF transmitter aided in recapturing marten, but was not used to obtain location data. Once we were satisfied with collar fit, we discontinued anesthesia and measured body mass using a wool toque and a spring scale. We then released the marten to its place of capture after a recovery time of 10–15 min. We recaptured marten and recovered their collars at the end of each winter field season using the same procedure as above.

### Analyses

*Mapping*—We mapped burn perimeters and severities in ArcMap using shapefiles from the USGS Geosciences and Environmental Change Science Center [68] and DataBC [69]. Burn severity layers were provided by the Monitoring Trends in Burn Severity project and BC Ministry of Forests, Lands, Natural Resource Operations and Rural Development. Mapping of the 2006 and 2010 burns used the differenced Normalized Burn Ratio (dNBR) [70], whereas the 2017 burn used the more recent Relativized Burn Ratio (RBR) [71]. Although the Relativized Burn Ratio produces a slightly better correlation to field measurements of burn severity, in practice the two metrics have a ~ 2% difference in accuracy [71]. We analyzed our burn severity layers as rasters with a minimum resolution of 30 × 30 m (0.09 ha). Burn severity classes for these data, based on surface reflectance at ~ 12 months post-fire for each raster cell, were (1) “unchanged” if no post-fire vegetation change was evident, (2) “low” for < 10% overstory tree mortality, (3) “moderate” for 10–70% mortality, or (4) “high” for > 70% mortality (Table 1) [70].

**Table 1:** Distribution of burn severities on post-fire landscapes in this study

Burn	Burn severity (%)				
	Unchanged	Low	Moderate	High	No Data
*Washington*					
2006	12.0	22.2	25.1	39.6	1.2
*British Columbia*					
2010	16.8	44.7	18.8	18.1	1.5
2017	45.2	27.8	27.0	0.1	0.0

Data are derived from the differenced Normalized Burn Ratio (2006 and 2010 burns) and Relativized Burn Ratio (2017 burn): unchanged = no post-fire vegetation change was evident, low =  < 10% overstory tree mortality, moderate = 10–70% mortality, and high =  > 70% mortality (Key and Benson 2006)

We generated custom shapefiles for roads, meadows, and salvage-logged areas using Landsat imagery and maps of 25-year landscape change from the National Forest Information System [3, 72]. The BC study area has a complex history of timber harvest beyond the scope of this study, so we considered treed areas of the 2010 and 2017 burns to be “intact forest,” even if some historical thinning was evident in Landsat imagery. We obtained hydrology shapefiles (streams and lakes) from the Washington Geospatial Open Data Portal [73] and DataBC [74].

In a few cases, marten trails in the 2010 burn extended into the 2017 burn or vice versa. We assigned each trail to a single burn based on where the majority of waypoints fell. For areas of the 2010 burn that re-burned in 2017, we assigned waypoints to the more recent disturbance. We omitted two trails in the 2010 burn that were < 100 m long.

*Trail Analyses*—We used the R package “trajr” to calculate marten movement characteristics on each trail [75]. Sinuosity *S* expresses the amount of angular change over a given path length, with straight paths approaching *S* = 0 and convoluted paths approaching *S* = 1 [76]. We estimated sinuosity using corrected methods from Benhamou [77]. For marten, we expected trails in low-quality habitat to have lower sinuosity. For trails that crossed meadows or salvage-blocks, we used ArcMap to measure net displacement *D* and straight-line distance *L* for trail segments within these habitats. We then calculated the straightness index *D*/*L* for each crossing [78]. Highly straight paths approach *D*/*L* = 1, and we expected marten to behave this way when crossing open areas. In practice, sinuosity and straightness are inversely related to each other [77]; we chose the straightness index to characterize crossings because we lacked a sufficiently large sample of angular change for these trail segments. Because we repeatedly detected marten activity over winter in areas where we back-tracked, we assumed that each trail was an independent foraging bout from a resident animal—in other words, an animal familiar with the landscape rather than merely dispersing through it.

We also used the R package “adehabitat” to determine the extent to which each trail deviated from the null model of correlated random walk, i.e. trail “directedness” [79, 80]. A random walk is characteristic of marten searching suitable habitat for prey, whereas a directed walk implies movement through lower-quality habitat. For this procedure, we first generated 1000 random trails with the same step length, total distance, and turning angle distribution as the original trail (see Additional file 1: Figure S1). We then calculated the mean squared displacement of each random trail and compared this distribution to the mean squared displacement of the original trail using a Monte Carlo permutation test. By definition, a directed walk differs significantly from the null hypothesis of random movement and represents a categorically different behaviour [79]. We therefore considered trails with high directedness (Monte Carlo *P* < 0.05) to be instances where marten shifted their foraging behaviour in response to habitat quality; most (62%) of trails that we identified as directed walks had a Monte Carlo *P* < 0.01 (see Additional file 1: Figure S2).

We assessed habitat selection along marten trails in terms of post-fire landscape change (burn severity) and landscape heterogeneity. For each georeferenced point along a trail we assigned a dNBR/RBR raster value of 0 = unburned, 1 = unchanged, 2 = low, 3 = moderate, or 4 = high. To quantify landscape change, we estimated overall burn severity (“burn index”) following methods in Roberts et al. [81]. We first calculated the proportions of burn severity classes along each trail using ArcMap, then multiplied these proportions by their corresponding dNBR/RBR raster values (0 to 4, as above); our burn index was the sum of these multiplied values (see Additional file 1: Figure S3). This index ranged from 0 if the trail fell entirely in unburned areas to a maximum of 4 if it crossed areas burned entirely at high severity, with intermediate values indicating moderate or mixed severity.

We quantified landscape heterogeneity (“burn diversity”) for each trail as a total sum of squares, using the proportions of unburned and burned habitat classes calculated above. We rescaled the index to range from 0 if the trail crossed a single habitat type to a maximum of 1 if all habitat types occurred in equal proportion, using the equation:

1 $$\frac{C-\sum_{i=1}^{n}({{y}_{i}-y)}^{2}}{C}$$

where *C* is a scaling factor equal to the theoretical maximum sum of squares if one habitat type dominates among *n* available habitat types.

For each burn, we used Grubbs’ test to identify and remove significant outliers among explanatory variables [82], then used Pearson correlations to assess collinearity, retaining variables with pairwise R^2^ < 0.5. We used univariate beta regressions with the R package “betareg” to examine the influence of canopy closure, burn index, and burn diversity on trail sinuosity [83]. We used univariate logistic regressions to examine the influence of these three habitat predictors on trail directedness. We chose a Gaussian distribution for model fit based on inspection of quantile-comparison plots, and pooled our data across all years. We assessed statistical significance with post-hoc Wald tests.

*Home Range Analyses*—We examined marten home ranges as utilization distributions (“kernels”) using the R packages “trajr” [75], “adehabitatLT”, and “adehabitatHR” [80]. We defined each animal’s home range as the area enclosed by a 90% fixed kernel, and its core activity area as the area enclosed by a 50% fixed kernel, using the biased random bridge method [84, 85]. Compared to simple kernels [86], this method models animal movement as a biased random walk and accounts for time lag between successive locations, producing utilization distributions that are more sensitive to travel corridors. To reduce bias from locations where marten were inactive (i.e. resting sites), we defined 23.7 m as the minimum distance between successive locations (*L*_min_), based on the estimated location error for a stationary collar at a low-severity site in the 2010 burn (N = 4498 locations). We defined 12.5 h as the maximum time lag between successive locations (*T*_max_), which covered > 90% of locations for all animals regardless of fix rate [85]. We selected the diffusion parameter *D* and smoothing parameter *h*_min_ based on the total number of locations for each marten. We visualized home range contours and calculated home range overlap using ArcMap.

To assess marten habitat selection at the home range scale, we used ArcMap to generate an equal number of randomly-located points (“available” locations) within a convex hull fitted to the outermost locations of each animal (“used” locations). We then assigned the following habitat attributes to each used and available location: (1) cover type (intact forest, meadow, or post-fire salvage), (2) burn severity (unburned, unchanged, low, moderate, or high), (3) distance to the nearest open water (streams or lakes), (4) distance to the nearest natural meadow, (5) distance to the nearest road, and (6) distance to the nearest salvage-logged area for BC. We evaluated marten responses to cover type and burn severity for each animal separately using chi-square post-hoc tests [87]. Likewise, we used logistic regressions to determine individual selection or avoidance of roads, open water, meadows, and post-fire salvage. We use “selected” and “chose” interchangeably in the sections below to describe how marten responded to available habitat features [88].

## Results

Over three winters, we back-tracked 102 marten trails for 65.5 km. Individual trails were 70 to 2200 m long with 14 to 440 5 m steps. Our final dataset included 50 trails covering 24.3 km in Washington; in BC, we back-tracked 33 trails covering 25.2 km in the 2010 burn and 19 trails covering 16.0 km in the 2017 burn. We found marten trails in unburned habitat on only one occasion (2006 burn). Although it was not possible to assign trails to individual marten, the majority of back-tracking took place beyond the home ranges of our collared animals.

**Fig. 2:**
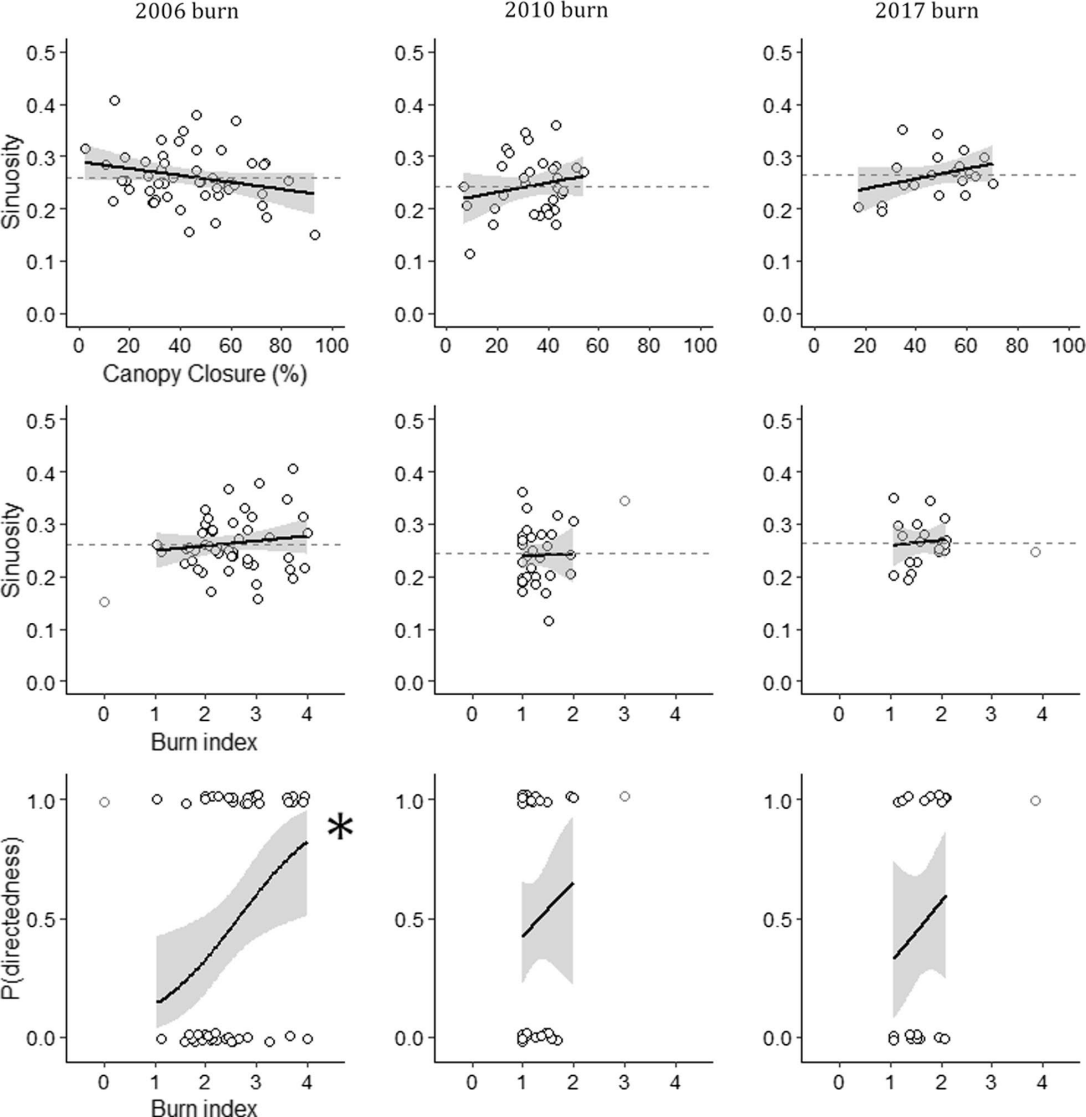
Marten movements in response to canopy closure and burn severity on post-fire landscapes, based on linear and logistic regressions. Data are from north-central Washington, USA (2006 burn), and central British Columbia, Canada (2010 and 2017 burns). “Burn index” measures the average burn severity along a marten trail from 0 (entirely unburned) to 4 (entirely high severity).Trails with high directedness, at y = 1, differ significantly from the characteristics of a correlated random walk, and would be expected from marten moving through low-quality habitats. Dashed lines show sample means; asterisks denote statistical significance. Light gray dots are significant outliers excluded from regressions. X-axis labels apply to all columns

Marten in the 2010 and 2017 burns did not significantly alter their movements in relation to canopy closure or burn index (Fig. 2). However, marten in the 2006 burn switched to directed movement in areas of high burn severity (N = 49, Z = 2.39, *P* = 0.017); they did not respond to canopy closure. Across all burns, marten did not alter their movements in relation to burn diversity (see Additional file 1: Figure S4).

Eight trails in BC crossed post-fire salvage-logged areas at least once, and three trails crossed natural meadows at least once (see Additional file 1: Table S2). Meadows were less common in the Washington study area, and we did not observe marten crossing them. Crossings averaged 197 ± 45 m through salvage-blocks (range 90–595 m, N = 11 crossings), and 267 ± 100 m in meadows (range 93–569 m, N = 5 crossings). Marten trails were highly straight in both habitat types, with *D*/*L* averaging 0.91 ± 0.04 in salvage-blocks (range 0.52–1.00, N = 11 crossings) and 0.89 ± 0.09 in meadows (range 0.53–1.00, N = 5 crossings).

**Fig. 3:**
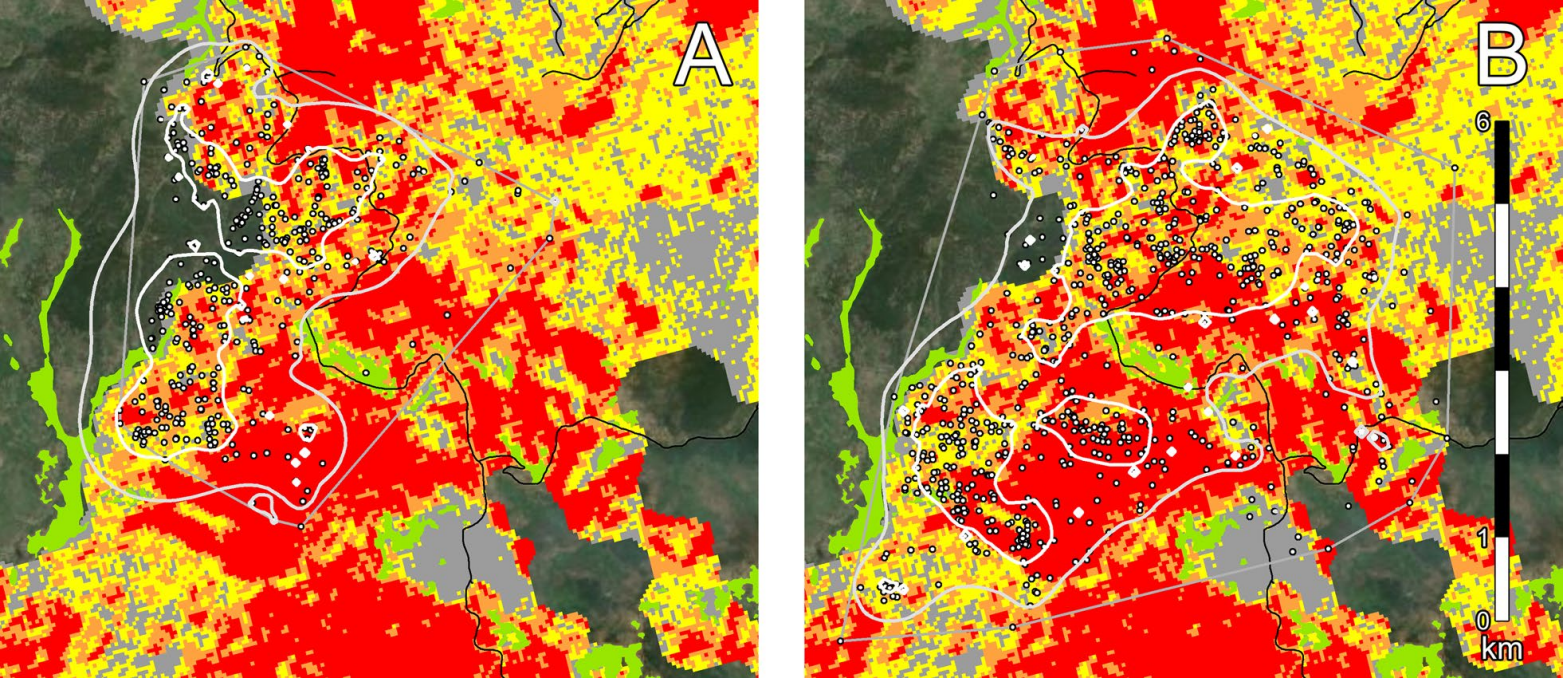
Marten locations post-fire in north-central Washington, USA (2006 burn), winter 2018–2019: **A** female WA-F1, and **B** male WA-M1. Solid lines denote 90% kernel home ranges (light gray) and 50% kernel core activity areas (white); thin gray lines denote convex hulls used in our analyses of habitat selection. Map colours are meadows (green) and burn severities: gray = unchanged (surface fires), yellow = low, orange = moderate, and red = high. Roads are shown in black

**Fig. 4:**
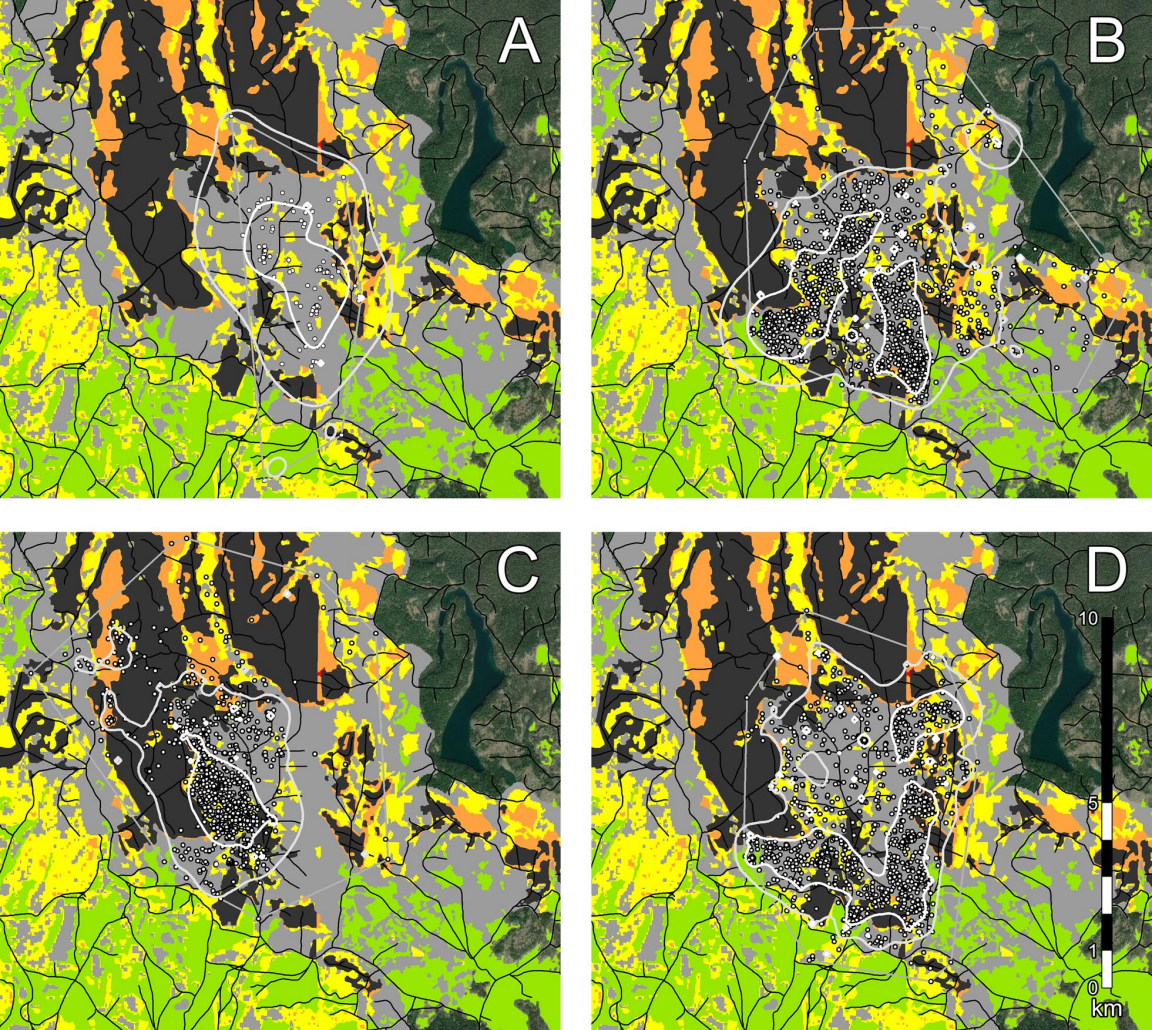
Marten locations post-fire in central British Columbia, Canada (2010 burn), winters 2016–2017 and 2017–2018:** A**) female BC-F1,** B**) male BC-M1,** C**) female BC-F2, and** D**) male BC-M2. Solid lines denote 90% kernel home ranges (light gray) and 50% kernel core activity areas (white); thin gray lines denote convex hulls used in our analyses of habitat selection. Map colours are meadows (green), post-fire salvage-logged areas (darkest gray), and burn severities: gray = unchanged (surface fires), yellow = low, orange = moderate, and red = high. Roads are shown in black

We obtained 6768 locations from six collared marten (Table 2; see Additional file 1: Table S3): 1187 locations from one male and one female marten in Washington (Fig. 3), and 5581 locations from two male and two female marten in BC (Fig. 4). Locations were primarily over winter (December–early March), but we also obtained locations after snowmelt (“summer”, late March–October) for one male marten in BC (see Additional file 1: Figure S5).

**Table 2:** Characteristics of marten home ranges on post-fire landscapes in north-central Washington, USA (2006 burn), and central British Columbia, Canada (2010 burn)

Marten ID	Dates collared	Fix rate (min)	N days	N locations	Home range (ha)	
					90%	50%
WA-F1	Jan. 16–Mar. 13, 2019	15	56	389	674.4	207.0
WA-M1	Jan. 13–Mar. 17, 2019	15	63	798	1223.3	398.6
BC-F1	Mar. 6–Mar. 25, 2017	90	19	117	1077.6	234.8
BC-F2	Dec. 17, 2017–Mar. 2, 2018	20	75	1181	777.2	153.6
BC-M1	Dec. 18, 2017–Mar. 3, 2018	20	75	1357	1354.3	375.3
BC-M2	Dec. 21, 2017–Mar. 4, 2018	20	73	1182	1345.8	414.4
	Mar. 9–Oct. 28, 2018	90	233	1744	1495.3	464.4

“Fix rate” refers to the time between location attempts. Male BC-M2 was re-collared for the March–October deployment

In both study areas, male marten covered 26–81% more of the post-fire landscape than females (Table 2). Males had winter home ranges averaging 1307.8 ± 42.3 ha, while females averaged 843.1 ± 121.0 ha. Males used core activity areas averaging 396.1 ± 11.4 ha, while female core areas averaged 198.5 ± 23.8 ha. Male BC-M2’s summer home range was similar in size to his winter home range (see Additional file 1: Figure S5).

Male and female marten had extensively overlapping home ranges in both study areas (Figs. 3 and 4). In the 2006 burn, 73.6% of female WA-F1’s home range was within male WA-M1’s home range, and 34.4% of her core activity area overlapped with the male’s core activity area. Similarly in the 2010 burn, 69.2% of female BC-F2’s home range was within male BC-M1’s home range, and 55.4% of her core activity area overlapped with this male’s core activity area; 69.2% of her home range was also within male BC-M2’s home range, but only 10.5% of her core activity area overlapped with this male’s core activity area.

Unexpectedly, male marten in BC had extensive winter home-range overlap: 75.8% of male BC-M1’s home range was within male BC-M2’s home range, and 53.6% of male BC-M1’s core activity area overlapped with the other male’s core activity area. Male BC-M2 used similar areas of the 2010 burn throughout the year; 78.0% of his summer home range overlapped with his winter home range, and 57.7% of his summer core activity area overlapped with his winter activity area (see Additional file 1: Figure S5).

**Fig. 5:**
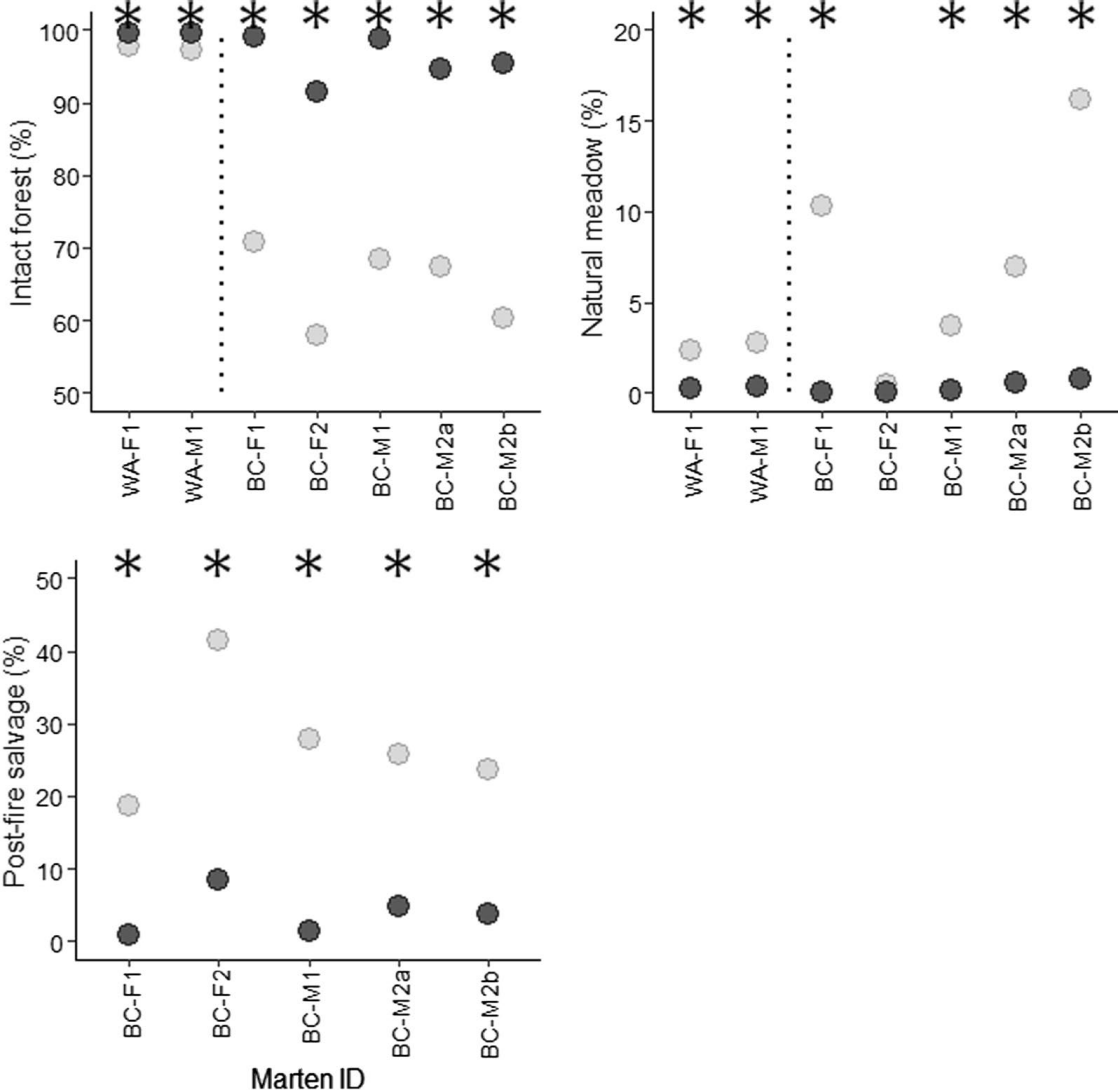
Marten home range composition, by cover type, on post-fire landscapes in north-central Washington, USA (2006 burn), and central British Columbia, Canada (2010 burn). Dark circles are marten locations (“used”) and light circles are random locations (“available”) within each animal’s home range envelope. “BC-M2a” and “BC-M2b” are winter and summer home ranges, respectively. Dotted lines separate the Washington and BC study areas. Asterisks denote statistical significance. Note different y-axis scales

Marten avoided open habitats (Fig. 5). Intact forest accounted for 91.5–99.7% of marten locations in winter (x̄ = 97.0%), and all marten used intact forest significantly more than expected from availability (x̄ = 76.6%). Natural meadows accounted for 0.0–0.6% of locations (x̄ = 0.2%), and five out of six marten used meadows areas significantly less than expected from availability (x̄ = 4.4%). In BC, 0.9–8.5% of winter locations occurred in salvage-logged areas. All marten used these areas significantly less than expected from availability (x̄ = 28.5%). Male BC-M2 had similar selection patterns in summer.

**Fig. 6:**
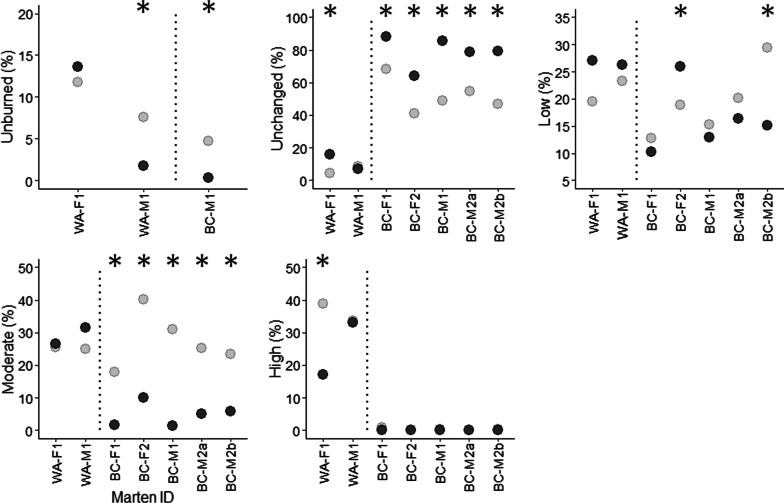
Marten home range composition, by burn severity, on post-fire landscapes in north-central Washington, USA (2006 burn), and central British Columbia, Canada (2010 burn). Dark circles are marten locations (“used”) and light circles are random locations (“available”) within each animal’s home range envelope. “BC-M2a” and “BC-M2b” are winter and summer home ranges, respectively. Dotted lines separate the Washington and BC study areas. Asterisks denote statistical significance. Note different y-axis scales

Marten responded to burn severity, with stronger patterns of selection in the 2010 burn than the 2006 burn (Fig. 6). In Washington, female WA-F1 used unchanged areas of the 2006 burn significantly more than expected (15.7 vs. 4.4%), and used high-severity areas significantly less than expected (17.2 vs. 38.8%). In BC, unchanged areas of the 2010 burn accounted 64.0–88.0% of all locations in winter (x̄ = 79.0%), and all marten used these areas significantly more than expected from availability (x̄ = 53.2%). Conversely, moderate-severity areas of the 2010 burn accounted for 1.5–10.1% of all locations (x̄ = 4.6%), and all marten used these areas significantly less than expected from availability (x̄ = 28.5%). Female BC-F2 used low-severity areas of the burn significantly more than expected (25.9 vs. 18.9%), but no other marten showed a significant response. Male BC-M2 had similar selection patterns in summer. Two animals with locations in unburned habitat (males WA-M1 and BC-M1), used unburned locations significantly less than expected (Fig. 6).

**Fig. 7:**
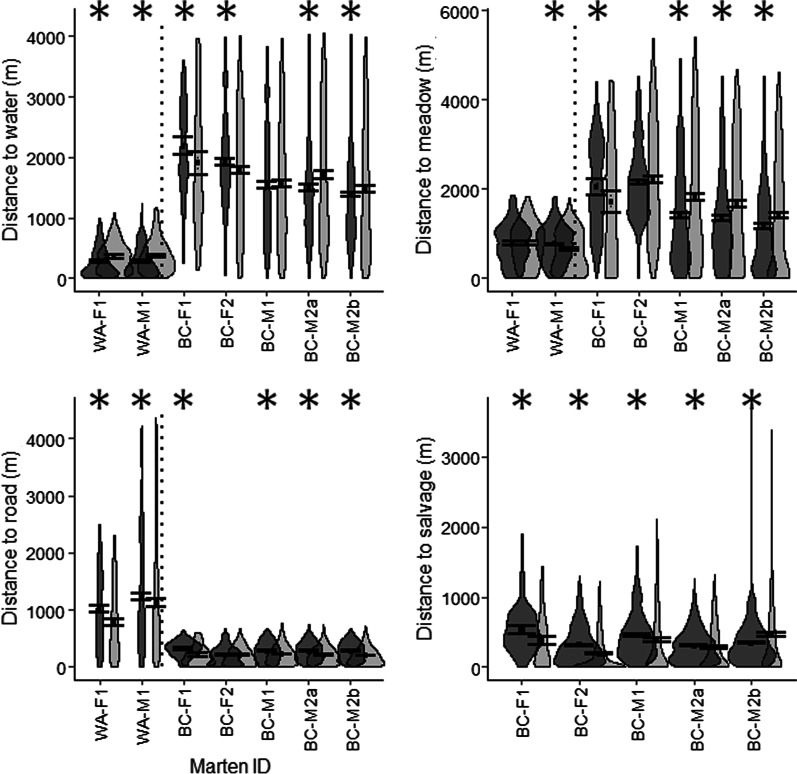
Marten home ranges in relation to habitat features on post-fire landscapes north-central Washington, USA (2006 burn), and central British Columbia, Canada (2010 burn). Violin plots show densities of marten locations (“used”; dark gray) versus random locations within each animal’s home range envelope (“available”; light gray). “BC-M2a” and “BC-M2b” are winter and summer home ranges, respectively. Horizontal bars denote bootstrapped means and 95% confidence intervals. Dotted lines separate the Washington and BC study areas; asterisks denote statistical significance. Note different y-axis scales

Individual marten responded differently to water, meadows, roads, and salvage-logged areas (Fig. 7; see Additional file 1: Table S4). Both marten in the 2006 burn selected areas closer to water compared to random locations, as did male BC-M2 in the 2010 burn (58–258 m closer; *P* < 0.01). Both females in the 2010 burn chose areas farther from water (86–421 m farther; *P* < 0.02) while male BC-M1 showed no significant patterns. Male WA-M1 in the 2006 burn and female BC-F1 in the 2010 burn chose areas farther from meadows (89–506 m farther; *P* < 0.05). Both males in the 2010 burn chose areas closer to meadows (198–461 m closer; *P* < 0.001), while female BC-F2 showed no significant patterns. All marten except female BC-F2 selected areas farther from roads (38–295 m farther, *P* < 0.05), and all marten in the 2010 burn chose areas farther from post-fire salvage in winter (13–210 m farther; *P* < 0.02). However, male BC-M2’s summer locations were significantly closer to these areas than expected (109–135 m closer; *P* < 0.001).

## Discussion

### Marten behaviour in burn mosaics

In both north-central Washington and central British Columbia, marten used a wide range of post-fire habitats but selected areas more similar to pre-fire conditions. As expected, marten were most active in areas with intact, residual trees and rarely used open meadows or post-fire salvage-logged areas. Marten altered their behaviour in response to habitat quality, adopting more directed movement through areas burned at high severity. In addition, marten chose home ranges that conspicuously excluded large patches of low-quality habitat.

Previous work has suggested that marten choose specific areas of burned landscapes. Trappers reported that marten were more abundant at the edges of young burns and areas burned at low severity [58]. Marten were most active along waterways and deadfall-rich areas seven years after fire in Alaska [50]. In the Northwest Territories, marten incorporated both burned and unburned habitats into their home ranges [51]. Marten evolved in fire-prone forests and clearly use heterogeneous post-fire landscapes [89]. Our work here indicates that large burns can support resident marten if portions of the landscape are relatively intact.

Local conditions influenced marten behaviour on burned landscapes. Marten in the 2006 burn made straighter movements in severely-burned habitats, where high-intensity fire caused substantial loss of trees, deadfall, and understory vegetation. Previous studies have shown that fine-scale habitat features affect marten movement [90, 91], and our work here indicates differences in path characteristics at larger spatial scales as well (200–2200 m). Areas of high burn severity have reduced overhead cover and less residual woody structure [19, 25]. Marten crossing these areas are more exposed to predators [56], and have fewer opportunities to access prey and shelter under snow [92]. Although marten sometimes foraged here, their tendency to beeline through open areas is consistent with optimal foraging theory, which predicts that animals minimize their activity in low-quality habitats [48, 93]. We did not see the same shift from random to directed movement from marten in younger burns, suggesting that long-term changes to the landscape may have a stronger negative influence on marten. Young burns contain more standing timber [94, 95], including damaged cone-bearing trees that may temporarily increase the suitability of severely-burned areas for marten and their prey. Red squirrel populations, for example, may not decline for several years post-disturbance depending on residual seed stores [96, 97].

Post-fire conditions influence marten home ranges as well. Marten in our study had home ranges similar in size to marten in burned black spruce (*Picea mariana*) in the Northwest Territories (11.1 km^2^; 21 years post-fire) [51], but roughly two times larger than marten home ranges in unburned mixed-conifer forests in California (2.3–8.1 km^2^) [98]. Male marten had larger home ranges than females, consistent with the larger size and higher energy requirements of males [98, 99]. Although our sample size is small and we did not collar marten in unburned areas, our results for the 2006 and 2010 burns are consistent with past work showing that marten need larger home ranges on low-quality landscapes [100, 101]. The high home range fidelity of male BC-M2 from winter to summer suggests that marten on burned landscapes need similarly large home ranges throughout the year, as has been seen on other disturbed landscapes [102].

We found substantial home range overlap for adult, resident males in the 2010 burn, which counters earlier statements that marten are territorial towards members of the same sex [98, 103]. This overlap may be a signature of landscape fragmentation forcing marten to share spatially limited resources. Coyotes (*Canis latrans*), for example, share remnant forest patches on agricultural landscapes [104]. Alternatively, male marten may have been competing for access to rare females, as has been documented in male-skewed populations of American badgers (*Taxidea taxus*) [105]. A larger sample of marten home ranges on burned landscapes would help determine which scenario is more likely.

Landscape context may have played a role in how marten responded to large-scale habitat features such as water and openings. Marten in the 2006 burn marten selected areas closer to water, but marten in the 2010 burn did not. Most water features in the 2006 burn were associated with steep drainages, and would have likely contained more deadfall and thicker post-fire vegetation than the surrounding hillsides [50]. In contrast, water features in the 2010 burn were typically lakes surrounded by gentle slopes. Although marten in both study areas avoided open habitats, marten in the 2010 burn did not respond to meadows and cut-blocks in the same way. These animals chose areas closer to natural meadows and farther from salvage-logged areas, suggesting a difference in habitat quality between the edges of meadows and the edges of cut-blocks [36].

Marten used all burn severities, but selected areas affected only by surface fires (unchanged) or burned at low severity. Our results agree with past work indicating the importance of these areas for marten [58], and support the view that marten will use severely-burned areas if enough residual structure is present [50]. Because burn severity reflects vegetation change [70], low-severity areas are more likely to retain habitat features suitable for marten: a closed canopy, large trees, and a structurally complex understory. Burn severity also shapes the distribution of marten prey including red-backed voles [59], red squirrels [60], western gray squirrels (*Sciurus griseus*) [106], and snowshoe hares [61]. Our detection of marten throughout all three burns is encouraging, as it suggests that even recent severely-burned areas can provide habitat structure and connectivity. In large burns, however, it seems likely that residual trees play a major role in determining where marten persist and establish home ranges.

Although marten choose habitats based on prey abundance and accessibility [57, 107], other factors may influence the placement and structure of marten home ranges. Marten may avoid areas of sparse overhead cover due to greater perceived predation risk [56, 108]. Notably, fishers sometimes prey on marten [109, 110] and select similar habitats; thus, competition with fishers in the highly fragmented BC study area may have influenced the distribution of resident marten [111, 112]. In addition, lynx, coyotes, wolves (*Canis lupus*), northern goshawks (*Accipiter gentilis*), and great horned owls (*Bubo virginianus*) could all potentially kill marten in our study areas if the opportunity arose. Tracking the activity patterns of other carnivores would help determine how marten factor predation risk into their habitat choices [113].

### Marten responses to salvage logging

**Fig. 8:**
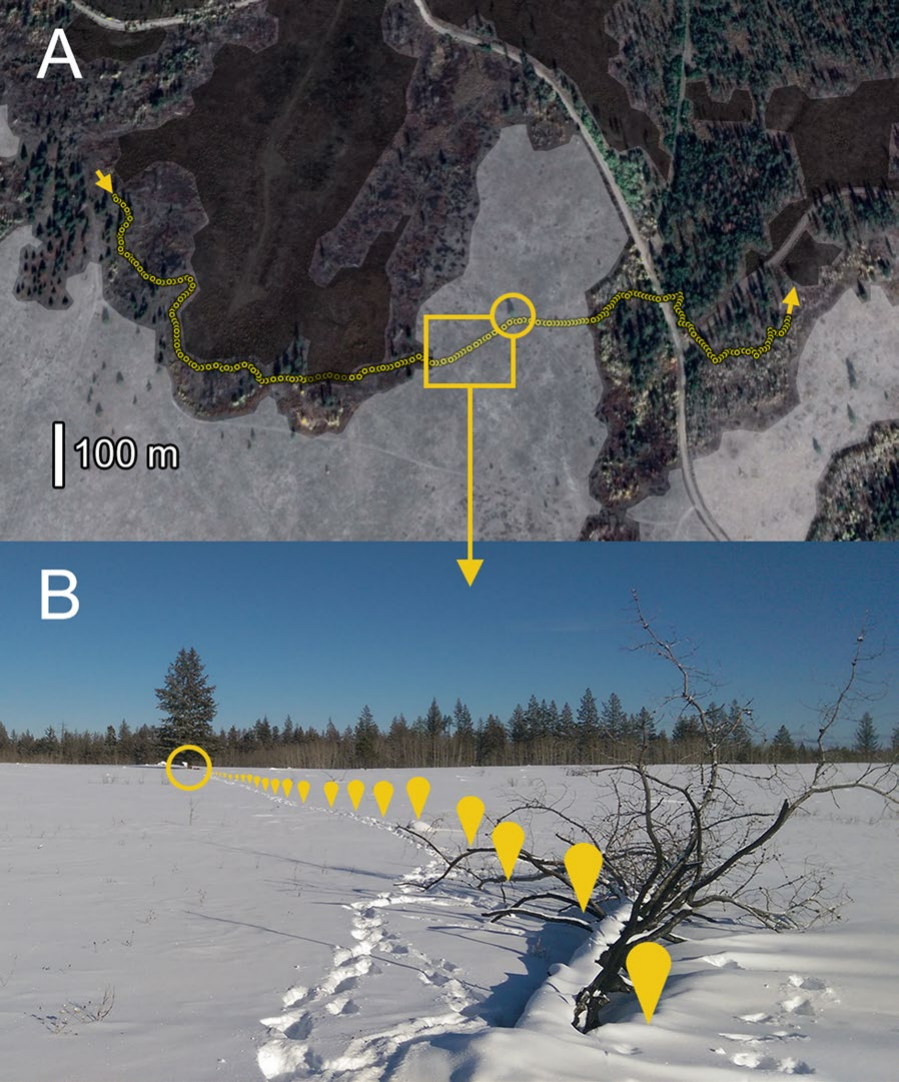
Marten use residual trees, snags, and deadfall as “stepping stones” to cross open habitats. **A** 5 m steps along a 1350 m trail in central British Columbia that burned in 2010 and 2017. **B** The trail segment and lone tree from the inset above (February 2019). Arrows at trail endpoints show the marten’s direction of movement. Natural meadows (light gray) and post-fire salvage-logged areas (dark gray) are low-quality habitats for marten; trail segments crossing these areas are essentially straight

Marten strongly avoided post-fire salvage-logged areas. Salvage-logged areas presented hard edges to marten home ranges that are clearly visible from mapped locations in the 2010 burn. Over three winters of snow-tracking, we rarely encountered marten trails that crossed salvage-logged areas or approached salvage-block edges. Remnant trees, saplings, and slash piles were important landmarks for marten crossing open habitats (Fig. 8), but these features were rare in salvage-logged areas. Marten were also less active near roads, which were extensive in and around salvage-logged areas of the 2010 burn.

We are not aware of any prior studies examining marten home ranges in salvage-logged areas. Indirectly, Steventon and Daust [114] modeled the potential impact of salvage logging on marten after large-scale beetle outbreaks in BC. This work forecast a substantial loss of suitable habitats for marten in the next 20–40 years due to landscape fragmentation, even if conventional timber harvest occurred at lower intensity. Because marten avoid natural openings [89, 115] and conventional clear-cuts [36, 47, 48], it is not surprising to see similar behaviour from marten in relation to salvage-logged areas.

Whether marten persist on burned landscapes depends on the quality of residual habitats: females need specific structures for denning [55, 116], whereas males need larger resource patches and larger prey [98, 117]. However, wildfire and salvage logging produce markedly different landscapes [65]. Low overhead cover and low structural complexity make salvage-logged areas unsuitable to both marten and their prey [39]. Road building in salvage-logged areas may also reduce the quality of nearby uncut stands, as it does in other managed forests [118]. In addition, salvage logging appears to sharply reduce connectivity between residual habitats, validating earlier forecasts of marten declines on these landscapes [114]. Although marten may tolerate a variety of post-fire conditions, salvage logging represents a cumulative disturbance that is substantially worse for marten than the original fire.

### Future Directions

Under climate change, fire regimes in western North American forests have shifted towards larger, more destructive, and more frequent patterns of burning [119–121]. These shifts are likely to continue [122, 123], and may result in the widespread replacement of forested landscapes with non-forest habitats by the end of the century [8–10]. In addition to habitat losses from fire, further fragmentation via post-fire salvage logging threatens biodiversity in burned forests [31–33]. In the face of such rapid landscape change, it is critical to understand how wildlife use burned landscapes [53, 124] and rethink forest management with fire in mind [125–127].

Although some forest wildlife is well-studied post-fire, important knowledge gaps remain for many species [53, 124, 128]. For marten, we recommend further work to understand the impacts of burn severity and salvage logging on landscape connectivity [129, 130] and population change [114]. Identifying where and when marten cross low-quality habitats would improve our understanding of marten movement ecology, and may help managers better emulate natural disturbance patterns on post-fire landscapes.

Given the limited scope and sample sizes in our study, we recommend further work to determine marten responses to fire and salvage logging more broadly in western forests. Large wildfires are inherently variable and non-replicable disturbances [131], and post-fire habitat conditions are not purely a function of time since fire [61, 132, 133]. Landscape-specific differences in topography, fire history, and management intensity, as well as year-to-year differences in regeneration, snowfall, and prey populations, all influence the conditions that marten encounter post-fire. Although we focus on marten behaviour in winter, when thermoregulation is more energetically costly [134], and deep snow makes foraging more difficult [135], we note that marten may perceive burned habitats differently at other times of year, such as late spring, when den trees become important for raising young [136]. Finally, although we found only modest correlations between habitat variables in our study, the combined effects of burn severity, time since fire, and habitat structure may influence marten behavior more strongly on other landscapes.

We believe that our results are most applicable to marten populations in recently-burned (< 15 years post-fire), pine-fir and spruce-fir forests in the Cascade Range [137] and Blue Mountains of Washington and Oregon [138], and in pine-fir forests of central and south-central BC [139]. We urge further work in boreal and mixed-conifer forests, such as those in the Sierra Nevada mountains, where fire behaviour and salvage logging practices differ.

The strong avoidance of salvage logging by marten raises concerns for other forest specialists. Like marten, female fishers use large, damaged trees and snags for denning [140] and males need large areas of dense forest [141], neither of which may be available on salvage-logged landscapes [142]. Fishers [143], Mexican fox squirrels (*Sciurus nayaritensis*) [144], black-backed woodpeckers (*Picoides arcticus*) [145], and American three-toed woodpeckers (*Picoides dorsalis*) [146] use forests burned at moderate to high severity, but these areas are often salvage-logged. Given the level of avoidance for salvage logging documented here, we recommend greater caution in post-fire landscape planning to protect habitats for wildlife. Residual treed areas are key areas for marten and other forest specialists, and their preservation should be a high priority.

## Conclusions

Our work provides further insight into marten behaviour on post-fire landscapes. Marten use recently-burned forests, but the inherent heterogeneity within burns strongly influences their habitat choices. Marten alter their movements in response to post-fire habitat quality; lightly-burned areas provide important residual structure for marten and offer suitable conditions for their preferred prey. However, marten avoid severely-burned areas and exclude salvage-blocks from their home ranges. Collectively, our results can inform management decisions that preserve marten habitat as fire and salvage logging change forested landscapes.

## Supplementary Information

**Additional file 1. Table S1.** Characteristics of post-fire landscapes examined in this study. **Table S2.** Marten movements when crossing post-fire salvage-logged areas and natural meadows. **Table S3.** Capture dates and physical characteristics of marten in this study. **Table S4.** Marten locations versus random locations in relation to stand-scale habitat features. **Figure S1.** Examples of marten movement behaviour in relation to habitat quality. **Figure S2.** Distribution of marten trails in our dataset along a continuum of trail “directedness”. **Figure S3.** Examples of "Burn index” measuring the overall post-fire burn severity on a portion of the landscape. **Figure S4.** Marten movements in response to post-fire canopy closure and landscape heterogeneity. **Figure S5.** Post-fire home range fidelity of male marten BC-M2.

## Data Availability

The datasets used and/or analysed during the current study are available from the corresponding author on reasonable request.

## Abbreviations

BC: British Columbia
dNBR: Differenced Normalized Burn Ratio
RBR: Relativized Burn Ratio

## References

[1] Curtis PG, Slay CM, Harris NL, Tyukavina A, Hansen MC (2018). Classifying drivers of global forest loss. Science.

[2] Cohen WB, Yang Z, Stehman SV, Schroeder TA, Bell DM, Masek JG, Huang C, Meigs GW (2016). Forest disturbance across the conterminous United States from 1985–2012: the emerging dominance of forest decline. For Ecol Manage.

[3] White JC, Wulder MA, Hermosilla T, Coops NC, Hobart GW (2017). A nationwide annual characterization of 25 years of forest disturbance and recovery for Canada using Landsat time series. Remote Sens Environ.

[4] Agee JK (1993). Fire ecology of Pacific Northwest forests.

[5] Stine P, Hessburg P, Spies T, Kramer M, Fettig CJ, Hansen A, Lehmkuhl J, O’Hara K, Polivka K, Singleton P, Charnley S, Merschel A, White R. The ecology and management of moist mixed-conifer forests in eastern Oregon and Washington: a synthesis of the relevant biophysical science and implications for future land management. Portland: USDA Forest Service, Pacific Northwest Research Station; 2014.

[6] Hessburg PF, Agee JK, Franklin JF (2005). Dry forests and wildland fires of the inland Northwest USA: Contrasting the landscape ecology of the pre-settlement and modern eras. For Ecol Manage.

[7] Miller JD, Safford HD, Crimmins M, Thode AE (2009). Quantitative evidence for increasing fire forest fire severity in the Sierra Nevada and southern Cascade Mountains, California and Nevada, USA. Ecosystems.

[8] Busby SU, Moffett KB, Holz A (2020). High-severity and short-interval wildfires limit forest recovery in the Central Cascade Range. Ecosphere.

[9] Westerling AL, Turner MG, Smithwick EAH, Romme WH, Ryan MG (2011). Continued warming could transform Greater Yellowstone fire regimes by mid-21st century. Proc Natl Acad Sci.

[10] Adams MA (2013). Mega-fires, tipping points and ecosystem services: managing forests and woodlands in an uncertain future. For Ecol Manage.

[11] Dolan KA, Hurtt GC, Flanagan SA, Fisk JP, Sahajpal R, Huang C, Page YL, Dubayah R, Masek JG (2017). Disturbance distance: quantifying forests’ vulnerability to disturbance under current and future conditions. Environ Res Lett.

[12] Sutherland EF, Dickman CR (1999). Mechanisms of recovery after fire by rodents in the Australian environment: a review. Wildl Res.

[13] Saab VA, Powell HDW (2005). Fire and avian ecology in North America: process influencing pattern. Stud Avian Biol.

[14] Fontaine JB, Kennedy PL (2012). Meta-analysis of avian and small-mammal response to fire severity and fire surrogate treatments in US fire-prone forests. Ecol Appl.

[15] Kotliar NB, Hejl SJ, Hutto RL, Saab VA, Melcher CP, McFadzen ME (2002). Effects of fire and post-fire salvage logging on avian communities in conifer-dominated forests of the western United States. Stud Avian Biol.

[16] Hossack BR, Pilliod DS (2011). Amphibian responses to wildfire in the western United States: emerging patterns from short-term studies. Fire Ecol.

[17] Fisher JT, Wilkinson L (2005). The response of mammals to forest fire and timber harvest in the North American boreal forest. Mammal Rev.

[18] Song SJ (2002). Ecological basis for stand management: a synthesis of ecological responses to wildfire and harvesting.

[19] Wood DJA, Drake S, Rushton SP, Rautenkranz D, Lurz PWW, Koprowski JL (2007). Fine-scale analysis of Mount Graham red squirrel habitat following disturbance. J Wildl Manag.

[20] Banks SC, Knight EJ, McBurney L, Blair D, Lindenmeyer DB (2011). The effects of wildfire on mortality and resources for an arboreal marsupial: resilience to fire events but susceptibility to fire regime change. PLoS ONE.

[21] Meehan TD, George TL (2003). Short-term effects of moderate- to high-severity wildfire on a disturbance-dependent flycatcher in northwest California. Auk.

[22] Covert-Bratland KA, Block WM, Theimer TC (2006). Hairy woodpecker winter ecology in ponderosa pine forests representing different ages since wildfire. J Wildl Manag.

[23] Simanonok MP, Burkle LA (2019). Nesting success of wood-cavity-nesting bees declines with increasing time since wildfire. Ecol Evol.

[24] Zielinski WJ, Schlexer FV (2019). The effect of time and forest disturbance on the structural and functional characteristics of fisher (*Pekania pennanti*) resting structures. Northwest Sci.

[25] Agee JK (1998). The landscape ecology of western forest fire regimes. Northwest Sci.

[26] Keeton WS, Franklin JF (2005). Do remnant old-growth trees accelerate rates of succession in mature Douglas-fir forests?. Ecol Monogr.

[27] Banks SC, Dujardin M, McBurney L, Blair D, Barker M, Lindenmayer DB (2011). Starting points for small mammal population recovery after wildfire: recolonization or residual populations?. Oikos.

[28] Steenvoorden J, Meddens AJH, Martinez AJ, Foster LJ, Kissling WD (2019). The potential importance of unburned islands as refugia for the persistence of wildlife species in fire-prone ecosystems. Ecol Evol.

[29] Dhar A, Parrott L, Hawkins CDB (2016). Aftermath of mountain pine beetle outbreak in British Columbia: stand dynamics, management response and ecosystem resilience. Forests.

[30] Franklin JF, Spies TA, Van Pelt R, Carey AB, Thornburgh DA, Berg DR, Lindenmayer DB, Harmon ME, Keeton WS, Shaw DC, Bible K, Chen J (2002). Disturbances and structural development of natural forest ecosystems with silvicultural implications, using Douglas-fir forests as an example. For Ecol Manage.

[31] Lindenmayer DB, Burton PJ, Franklin JF (2008). Salvage logging and its ecological consequences.

[32] Nappi A, Drapeau P, Savard JPL (2004). Salvage logging after wildfire in the boreal forest: is it becoming a hot issue for wildlife?. For Chron.

[33] Thorn S, Bässler C, Brandl R, Burton PJ, Cahall R, Campbell JL, Castro J, Choi CY, Cobb T, Durska E, Fontaine JB, Gauthier S, Hebert C, Hothorn T, Hutto RL, Lee EJ, Leverkus AB, Lindenmayer DB, Obrist MK, Rost J, Seibold S, Seidl R, Thom D, Waldron K, Wermelinger B, Winter MB, Zmihorski M, Müller J (2018). Impacts of salvage logging on biodiversity: a meta-analysis. J Appl Ecol.

[34] Koehler GM, Maletzke BT, Von Kienast JA, Aubry KB, Wielgus RB, Naney RH (2008). Habitat fragmentation and the persistence of lynx populations in Washington State. J Wildl Manag.

[35] Sauder JD, Rachlow JL (2014). Both forest composition and configuration influence landscape-scale habitat selection by fishers (*Pekania pennanti*) in mixed coniferous forests of the northern Rocky Mountains. For Ecol Manage.

[36] Hargis CD, Bissonette JA, Turner DL (1999). The influence of forest fragmentation and landscape pattern on American martens. J Appl Ecol.

[37] Smucker KM, Hutto RL, Steele BM (2005). Changes in bird abundance after wildfire: importance of fire severity and time since fire. Ecol Appl.

[38] Vanbianchi C, Gaines WL, Murphy MA, Hodges KA (2018). Navigating fragmented landscapes: Canada lynx brave poor quality habitats while traveling. Ecol Evol.

[39] Kelly AJ, Hodges KE (2020). Post-fire salvage logging reduces snowshoe hare and red squirrel densities in early seral stages. For Ecol Manage.

[40] Rockweit JT, Franklin AB, Carlson PC (2017). Differential impacts of wildfire on the population dynamics of an old-forest species. Ecology.

[41] O’Neil ST, Coates PS, Brussee BE, Ricca MA, Espinosa SP, Gardner SC, Delehanty DJ (2020). Wildfire and the ecological niche: diminishing habitat suitability for an indicator species within semi-arid ecosystems. Glob Change Biol.

[42] Patten MA, Kelly JF (2010). Habitat selection and the perceptual trap. Ecol Appl.

[43] Buskirk SW, Powell RA, Buskirk SW, Harestad AS, Raphael MG, Powell RA (1994). Habitat ecology of fishers and American martens. Martens, sables, and fishers: biology and conservation.

[44] Thompson ID, Fryxell J, Harrison DJ, Aubry KB, Zielinski WJ, Raphael MG, Proulx G, Buskirk SW (2012). Improved insights into use of habitat by American martens. Biology and conservation of martens, sables, and fishers: a new synthesis.

[45] Watt WR, Baker JA, Hogg DM, McNicol JG, Naylor BJ. Forest management guidelines for the provision of marten habitat. Version 1.0. Sault Ste. Marie: Ontario Ministry of Natural Resources; 1996.

[46] Guppy CS. Guide to species of management concern. Report prepared for BC Timber Sales, Skeena Business Area. Terrace: BC Ministry of Forests and Range; 2008.

[47] Soutiere EC (1979). Effects of timber harvesting on marten in Maine. J Wildl Manag.

[48] Cushman SA, Raphael MG, Ruggiero LF, Shirk AS, Wasserman TN, O’Doherty EC (2011). Limiting factors and landscape connectivity: the American marten in the Rocky Mountains. Landsc Ecol.

[49] Raine RM (1982). Ranges of juvenile fisher, *Martes pennanti*, and marten, *Martes americana*, in southeastern Manitoba. Can Field-Nat.

[50] Magoun AJ, Vernam DJ. An evaluation of the Bear Creek burn as marten (*Martes americana*) habitat in interior Alaska. Final Report, Special Cooperative Project AK-950-CAH-0. Fairbanks: US Bureau of Land Management and Alaska Department of Fish and Game; 1986.

[51] Latour PB, Maclean N, Poole KG (1994). Movements of martens, *Martes americana*, in burned and unburned taiga in the Mackenzie Valley, Northwest Territories. Can Field-Nat.

[52] Paragi TF, Johnson WN, Katnik DD, Magoun AJ (1996). Marten selection of postfire seres in the Alaskan taiga. Can J Zool.

[53] Volkmann LA, Hutchen J, Hodges KE (2020). Trends in carnivore and ungulate fire ecology research in North American conifer forests. For Ecol Manage.

[54] Buskirk SW, Forrest SC, Raphael MG, Harlow HJ (1989). Winter resting site ecology of marten in the central Rocky Mountains. J Wildl Manag.

[55] Ruggiero LF, Pearson DE, Henry SE (1998). Characteristics of American marten den sites in Wyoming. J Wildl Manag.

[56] Herman T, Fuller K (1974). Observations of the marten, *Martes americana*, in the Mackenzie District, Northwest Territories. Can Field-Nat.

[57] Andruskiw M, Fryxell JM, Thompson ID, Baker JA (2008). Habitat-mediated variation in predation risk by the American marten. Ecology.

[58] Stephenson RO. The relationship of fire history to furbearer populations and harvest. Final Report, Project W-22-2, Job 7.13 R. Juneau: Alaska Department of Fish and Game; 1984.

[59] Zwolak R, Foresman KR (2007). Effects of a stand-replacing fire on small-mammal communities in montane forest. Can J Zool.

[60] Podruzny SR, Reinhart DP, Mattson DJ (1999). Fire, red squirrels, whitebark pine, and Yellowstone grizzly bears. Ursus.

[61] Hutchen J, Hodges KE (2019). Impact of wildfire size on snowshoe hare relative abundance in southern British Columbia, Canada. Fire Ecol.

[62] Thomas JP, Reid ML, Barlcay RMR, Jung TS (2019). Salvage logging after an insect outbreak reduces occupancy by snowshoe hares (*Lepus americanus*) and their primary predators. Global Ecol Conserv.

[63] Kelly A. Small mammals and mesomammals in a post-fire and salvage-logged landscape. MSc thesis. Kelowna: University of British Columbia Okanagan; 2021.

[64] Hope GD, Mitchell WR, Lloyd DA, Erickson WR, Harper WL, Wikeem BM, Meidinger D, Pojar J (1991). Interior Douglas-fir zone. Ecosystems of British Columbia Special Report Series 6.

[65] Volkmann LA. Habitat selection by Pacific marten (*Martes Caurina*) and other carnivores after wildfire and post-fire salvage logging. Ph.D. dissertation. Kelowna: University of British Columbia Okanagan; 2021.

[66] Bull EL, Heater TW, Culver FG (1996). Live-trapping and immobilizing American martens. Wildl Soc Bull.

[67] Desmarchelier M, Cheveau M, Imbeau L, Lair S (2007). Field use of isoflurane as an inhalant anesthetic in the American marten (*Martes americana*). J Wildl Dis.

[68] USGS Geosciences and Environmental Change Science Center: GeoMAC. http://rmgsc.cr.usgs.gov/outgoing/GeoMAC. Accessed 22 June 2020.

[69] BC Wildfire Service: Fire Perimeters – Historical. https://catalogue.data.gov.bc.ca/dataset/fire-perimeters-historical. Accessed 22 June 2020.

[70] Key CH, Benson NC. Landscape Assessment: ground measure of severity, the Composite Burn Index; and remote sensing of severity, the Normalized Burn Ratio. In Lutes DC, Keane RE, Caratti JF, Key CH, Benson NC, Sutherland S, Gangi LJ, editors. FIREMON: fire effects monitoring and inventory system. General Technical Report RMRS-GTR-164-CD: LA 1–51. Ogden: USDA Forest Service; 2006. p. 1–51.

[71] Parks SA, Dillon GK, Miller C (2014). A new metric for quantifying burn severity: the relativized burn ratio. Remote Sensing.

[72] Government of Canada: High Resolution Forest Change for Canada (Change Year) 1985–2011. https://open.canada.ca/data/en/dataset/5a316fdc-3237-4ace-831e-67b4ca26a248. Accessed 22 June 2020.

[73] Washington Geospatial Open Data Portal: WA Hydrography - NHD Flowline. https://geo.wa.gov/datasets/waecy::wa-hydrography-nhd-flowline. Accessed 22 June 2020.

[74] Geo BC: Freshwater Atlas Stream Network. https://catalogue.data.gov.bc.ca/dataset/freshwater-atlas-stream-network. Accessed 22 June 2020.

[75] McLean DJ, Skowron Volponi MA (2018). trajr: an R package for characterisation of animal trajectories. Ethol Methods.

[76] Bovet P, Benhamou S (1988). Spatial analysis of animals’ movements using a correlated random walk model. J Theor Biol.

[77] Benhamou S (2004). How to reliably estimate the tortuosity of an animal’s path: straightness, sinuosity, or fractal dimension?. J Theor Biol.

[78] Batschelet E (1981). Circular statistics in biology.

[79] Kareiva PM, Shigesada N (1983). Analyzing insect movement as a correlated random walk. Oecologia.

[80] Calenge C (2006). The package “adehabitat” for the R software: a tool for the analysis of space and habitat use by animals. Ecol Model.

[81] Roberts SL, van Wagtendonk JW, Miles AK, Kelt DA, Lutz JA (2008). Modeling the effects of fire severity and spatial complexity on small mammals in Yosemite National Park, California. Fire Ecol.

[82] Grubbs FE (1950). Sample criteria for testing outlying observations. Ann Math Stat.

[83] Cribari-Neto F, Zeileis A (2010). Beta regression in R. J Stat Softw.

[84] Benhamou S, Cornelis D (2010). Incorporating movement behavior and barriers to improve biological relevance of kernel home range space use estimates. J Wildl Manag.

[85] Benhamou S (2011). Dynamic approach to space and habitat use based on biased random bridges. PLoS ONE.

[86] Worton BJ (1989). Kernel methods for estimating the utilization distribution in home-range studies. Ecology.

[87] Beasley TM, Schumacker RE (1995). Multiple regression approach to analyzing contingency tables: post hoc and planned comparison procedures. J Exp Educ.

[88] Hall LS, Krausman PR, Morrison ML (1997). The habitat concept and a plea for standard terminology. Wildl Soc Bull.

[89] Koehler GM, Hornocker MG (1977). Fire effects on marten habitat in the Selway-Bitterroot Wilderness. J Wildl Manag.

[90] Nams VO, Bourgeois M (2004). Fractal analysis measures habitat use at different spatial scales: an example with American marten. Can J Zool.

[91] Vigeant-Langlois C, Desrochers A (2011). Movements of wintering American martens (*Martes americana*): relative influences of prey activity and forest stand age. Can J For Res.

[92] Corn JG, Raphael MG (1992). Habitat characteristics at marten subnivean access sites. J Wildl Manag.

[93] Charnov EL (1976). Optimal foraging: the marginal value theorem. Theor Popul Biol.

[94] Harper KA, Bergeron Y, Drapeau P, Gauthier S, De Grandpré L (2005). Structural development following fire in black spruce boreal forest. For Ecol Manage.

[95] Grayson LM, Cluck DR, Hood SM (2019). Persistence of fire-killed conifer snags in California, USA. Fire Ecology.

[96] Wheatley M, Larsen KW, Boutin S (2002). Does density reflect habitat quality for North American red squirrels during a spruce-cone failure?. J Mammal.

[97] Herbers J, Klenner W (2007). Effects of logging pattern and intensity on squirrel demography. J Wildl Manag.

[98] Powell RA, Buskirk SW, Harestad AS, Raphael MG, Powell RA (1994). Structure and spacing of *Martes* populations. Martens, sables, and fishers: biology and conservation.

[99] Buskirk SW, McDonald LL (1989). Analysis of variability in home-range size of the American marten. J Wildl Manag.

[100] Fuller AK, Harrison DJ (2005). Influence of partial timber harvesting on American martens in north-central Maine. J Wildl Manag.

[101] Gosse JW, Cox R, Avery SW (2005). Home-range characteristics and habitat use by American martens in eastern Newfoundland. J Mammal.

[102] Phillips DM, Harrison DJ, Payer DC (1998). Seasonal changes in home-range area and fidelity of martens. J Mammal.

[103] Bull EL, Heater TW (2001). Home range and dispersal of the American marten in northeastern Oregon. Northwest Nat.

[104] Atwood TC, Weeks HP (2003). Spatial home-range overlap and temporal interaction in eastern coyotes: the influence of pair types and fragmentation. Can J Zool.

[105] Minta SC (1993). Sexual differences in spatio-temporal interaction among badgers. Oecologia.

[106] Mazzamuto MV, Mazzella MN, Merrick MJ, Koprowski JL (2020). Fire impacts on a forest obligate: western gray squirrel response to burn severity. Mamm Biol.

[107] Coffin CW, Kujala QJ, Douglass RJ, Irby LR, Proulx G, Bryant HN, Woodward PM (1997). Interactions among marten prey availability, vulnerability, and habitat structure. *Martes*: taxonomy, ecology, techniques, and management.

[108] Kautz TM, Beyer DE, Farley Z, Fowler NL, Kellner KF, Lutto AL, Petroelje TR, Belant JL (2021). American martens use vigilance and short-term avoidance to navigate a landscape of fear from fishers at artificial scavenging sites. Sci Rep.

[109] Raine MR (1987). Winter food habits and foraging behaviour of fishers (*Martes pennanti*) and martens (*Martes americana*) in southeastern Manitoba. Can J Zool.

[110] Zielinski WJ, Duncan NP, Farmer EC, Truex RL, Clevenger AP, Barrett RH (1999). Diet of fishers (*Martes pennanti*) at the southernmost extent of their range. J Mammal.

[111] Fisher JT, Anholt B, Bradbury S, Wheatley M, Volpe JP (2013). Spatial segregation of sympatric marten and fishers: the influence of landscapes and species-scapes. Ecography.

[112] Manlick PJ, Woodford JE, Zuckerberg B, Pauli JN (2017). Niche compression intensifies competition between reintroduced American martens (*Martes americana*) and fishers (*Pekania pennanti*). J Mammal.

[113] Kemna CJ, Nagy-Reis MB, Scrafford MA (2010). Temporal segregation among sympatric boreal predators. Mammal Res.

[114] Steventon JD, Daust DK (2009). Management strategies for a large-scale mountain pine beetle outbreak: modelling impacts on American martens. For Ecol Manage.

[115] Spencer WD, Barrett RH, Zielinski WJ (1983). Marten habitat preferences in the northern Sierra Nevada. J Wildl Manag.

[116] Wynne KM, Sherburne JA (1984). Summer home range use by adult marten in northwestern Maine. Can J Zool.

[117] Chapin TG, Harrison DJ, Katnik DD (1998). Influence of landscape pattern on habitat use by American marten in an industrial forest. Conserv Biol.

[118] Robitaille JF, Aubry K (2000). Occurrence and activity of American martens *Martes americana* in relation to roads and other routes. Acta Theriol.

[119] Kasischke ES, Turetsky MR (2006). Recent changes in the fire regime across the North American boreal region—spatial and temporal patterns of burning across Canada and Alaska. Geophys Res Lett.

[120] Westerling AL, Hidalgo HG, Cayan DR, Swetnam TW (2006). Warming and earlier spring increase western US forest wildfire activity. Science.

[121] Dennison PE, Brewer SC, Arnold JD, Moritz MA (2014). Large wildfire trends in the western United States, 1984–2011. Geophys Res Lett.

[122] McKenzie DS, Gedalof ZE, Peterson DL, Mote P (2004). Climate change, wildfire, and conservation. Conserv Biol.

[123] Girardin MP, Mudelsee M (2008). Past and future changes in Canadian boreal wildfire activity. Ecol Appl.

[124] Hutchen J, Volkmann LA, Hodges KE (2017). Experimental designs for studying small-mammal responses to fire in North American conifer forests. Int J Wildland Fire.

[125] Hutto RL (2006). Toward meaningful snag-management guidelines for postfire salvage logging in North American conifer forests. Conserv Biol.

[126] Millar CI, Stephenson NL, Stephens SL (2007). Climate change and forests of the future: managing in the face of uncertainty. Ecol Appl.

[127] Lindenmayer DB, Laurence WF, Franklin JF, Likens GE, Banks SC, Blanchard W, Gibbons P, Ikin K, Blair D, McBurney L, Manning AD, Stein JAR (2014). New policies for old trees: averting a global crisis in a keystone ecological structure. Conserv Lett.

[128] Geary WL, Doherty TS, Nimmo DG, Tulloch AIT, Ritchie EG (2020). Predator responses to fire: a global systematic review and meta-analysis. J Anim Ecol.

[129] Moriarty KM, Epps CW, Betts MG, Hance DJ, Bailey JD, Zielinski WJ (2015). Experimental evidence that simplified forest structure interacts with snow cover to influence functional connectivity for Pacific martens. Landscape Ecol.

[130] Seip CR, Hodder DP, Crowley SM, Johnson CJ (2018). Use of constructed coarse woody debris corridors in a clearcut by American martens (*Martes americana*) and their prey. Forestry.

[131] Fraterrigo JM, Rusak JA (2008). Disturbance-driven changes in the variability of ecological patterns and processes. Ecol Lett.

[132] Swan M, Christie F, Sitters H, York A, Di Stefano J (2015). Predicting faunal fire responses in heterogeneous landscapes: the role of habitat structure. Ecol Appl.

[133] Vanbianchi CM, Murphy MA, Hodges KE (2017). Canada lynx use of burned areas: conservation implications of changing fire regimes. Ecol Evol.

[134] Buskirk SW, Harlow HJ (1989). Body-fat dynamics of the American marten (*Martes americana*) in winter. J Mammal.

[135] Wilbert CJ, Buskirk SW, Gerow KG (2000). Effects of weather and snow on habitat selection by American martens (*Martes americana*). Can J Zool.

[136] Godbout G, Ouellet JP (2010). Habitat selection of American marten in a logged landscape at the southern fringe of the boreal forest. Écoscience.

[137] Koehler GM, Blakesley JA, Koehler TW (1990). Marten use of successional forest stages during winter in north-central Washington. Northwest Nat.

[138] Bull EL, Heater TW, Shepherd JF (2005). Habitat selection by the American marten in northeastern Oregon. Northwest Sci.

[139] Mowat G (2006). Winter habitat associations of American martens *Martes americana* in interior wet-belt forests. Wildl Biol.

[140] Weir RD, Phinney M, Lofroth EC (2012). Big, sick, and rotting: why tree size, damage, and decay are important to fisher reproductive habitat. For Ecol Manage.

[141] Zielinski WJ, Truex RL, Schmidt GA, Schlexer FV, Schmidt KN, Barrett RH (2004). Home range characteristics of fishers in California. J Mammal.

[142] Lindenmayer DB, Blanchard W, McBurney L, Blair D, Banks S, Likens GE, Franklin JF, Laurance WF, Stein JAR, Gibbons P (2012). Interacting factors driving a major loss of large trees with cavities in a forest ecosystem. PLoS ONE.

[143] Hanson CT (2015). Use of higher severity fire areas by female Pacific fishers on the Kern Plateau, Sierra Nevada, California, USA. Wildl Soc Bull.

[144] Doumas SL, Koprowski JL (2013). Effect of heterogeneity in burn severity on Mexican fox squirrels following the return of fire. Int J Wildland Fire.

[145] Koivula MJ, Schmiegelow FKA (2007). Boreal woodpecker assemblages in recently burned forested landscapes in Alberta, Canada: effects of post-fire harvesting and burn severity. For Ecol Manage.

[146] Kotliar NB, Reynolds EW, Deutschman DH (2008). American three-toed woodpecker response to burn severity and prey availability at multiple spatial scales. Fire Ecol.

