# Supplementary material for: Post-fire movements of Pacific marten (Martes caurina) depend on the severity of landscape change
Source: Mov Ecol. 2021 Oct 9;9:49. doi: 10.1186/s40462-021-00286-2 (PMC8501742; doi:10.1186/s40462-021-00286-2)
Supplement: Supplementary file 1 — Additional file 1. Table S1. Characteristics of post-fire landscapes examined in this study. Table S2. Marten movements when crossing post-fire salvage-logged areas and natural meadows. Table S3. Capture dates and physical characteristics of marten in this study. Table S4. Marten locations versus random locations in relation to stand-scale habitat features. Figure S1. Examples of marten movement behaviour in relation to habitat quality. Figure S2. Distribution of marten trails in our dataset along a continuum of trail “directedness”. Figure S3. Examples of "Burn index” measuring the overall post-fire burn severity on a portion of the landscape. Figure S4. Marten movements in response to post-fire canopy closure and landscape heterogeneity. Figure S5. Post-fire home range fidelity of male marten BC-M2. [file 40462_2021_286_MOESM1_ESM.docx]

**Additional File 1**

**Table S1.** Characteristics of post-fire landscapes examined in this study. All three fires were lightning-caused.

| Wildfire name | | Year | Date discovered | Area burned (ha) | Burn perimeter (km) | Post-fire salvage logging |
| --- | --- | --- | --- | --- | --- | --- |
| *Washington* | |  |  |  |  |  |
|  | Tripod | 2006 | July 24 | 70,575 | 770.9 | none |
| *British Columbia* | |  |  |  |  |  |
|  | Meldrum Creek | 2010 | July 27 | 15,553 | 82.5 | 2010-2012 |
|  | Hanceville-Riske Creek | 2017 | July 7 | 239,340 | 575.6 | 2017-2019 |

**Table S2.** Marten movements when crossing post-fire salvage-logged areas and natural meadows in central British Columbia, Canada (2010 and 2017 burns). The straightness index *D*/*L* expresses net displacement *D* over straight-line distance *L*, with highly straight trails approaching *D*/*L* = 1. Salvage-logged areas were harvested following the 2010 burn.

| Trail ID | | Track Date | N waypoints  in crossing | *D* (m) | *L* (m) | *D/L* |
| --- | --- | --- | --- | --- | --- | --- |
| *Post-fire salvage* | |  |  |  |  |  |
|  | 2010-1.8 | 14 Feb., 2017 | 4 | 136 | 144 | 0.94 |
|  | 2010-1.11 | 23 Feb., 2017 | 14 | 470 | 595 | 0.79 |
|  | 2010-2.5 | 19 Jan., 2018 | 4 | 130 | 133 | 0.98 |
|  | 2010-3.10 | 20 Jan., 2019 | 4 | 138 | 141 | 0.98 |
|  |  |  | 4 | 138 | 138 | 1.00 |
|  | 2010-3.11 | 21 Jan., 2019 | 8 | 317 | 333 | 0.95 |
|  | 2017-3.7 | 16 Jan., 2019 | 3 | 90 | 90 | 1.00 |
|  | 2017-3.15 | 1 Feb., 2019 | 3 | 95 | 96 | 0.99 |
|  |  |  | 6 | 212 | 230 | 0.92 |
|  | 2017-3.17 | 13 Feb., 2019 | 4 | 68 | 131 | 0.52 |
|  |  |  | 4 | 130 | 132 | 0.98 |
| *Meadows* | |  |  |  |  |  |
|  | 2017-2.3 | 16 Jan., 2018 | 3 | 91 | 93 | 0.98 |
|  |  |  | 3 | 93 | 93 | 1.00 |
|  |  |  | 14 | 299 | 569 | 0.53 |
|  | 2017-2.8 | 22 Jan., 2018 | 4 | 137 | 138 | 0.99 |
|  | 2017-3.17 | 13 Feb., 2019 | 10 | 423 | 441 | 0.96 |

**Table S3.** Capture dates and physical characteristics of marten in this study.

|  |  |  |  | GPS collars | | |
| --- | --- | --- | --- | --- | --- | --- |
| Marten ID | | Sex | Body mass (g) | Collar ID | Date deployed | Date recovered |
| *Washington* | | |  |  |  |  |
|  | WA-F1 | F | 705 | 1444 | Jan. 16, 2019 | Mar. 13, 2018 |
|  | WA-F2 | F | 630 | 1392 | Jan. 28, 2019 | — |
|  | WA-M1 | M | 960 | 1437 | Jan. 13, 2019 | Mar. 17, 2018 |
|  | WA-M2 | M | 960 | 1451 | Jan. 26, 2019 | — |
|  | WA-M3 | M | 1100 | 1412 | Jan. 29, 2019 | — |
| *British Columbia* | | |  |  |  |  |
|  | BC-F1 | F | 750 | 1440 | Mar. 6, 2017 | Mar. 16, 2018 |
|  | BC-F2 | F | 760 | 1445 | Dec. 17,2017 | Mar. 2, 2018 |
|  |  |  | 760 | 1441 | Mar. 2, 2018 | — |
|  | BC-M1 | M | 980 | 1408 | Dec. 18,2017 | Mar. 3, 2018 |
|  |  |  | 1100 | 1345 | Mar. 3, 2018 | — |
|  | BC-M2 | M | 1100 | 1437 | Dec. 21,2017 | Mar. 4, 2018 |
|  |  |  | 1220 | 1324 | Mar. 4, 2018 | Feb. 28, 2019 |
|  | BC-M3 | M | 1100 | 1404 | Mar. 7, 2017 | — |

**Table S4.** Marten locations (“used”) versus random locations (“available”) in relation to stand-scale habitat features on post-fire landscapes in north-central Washington , USA (2006 burn), and central British Columbia, Canada (2010 burn). Means and confidence intervals are bootstrapped values. Bold entries indicate significant selection or avoidance by marten, based on single-factor logistic regressions.

| Marten ID | | Used | | Available | | Estimate | SE | Z | P |
| --- | --- | --- | --- | --- | --- | --- | --- | --- | --- |
|  |  | $\bar{x}$ | 95% CI | $\bar{x}$ | 95% CI |  |  |  |  |
| *Distance to water* | |  |  |  |  |  |  |  |  |
|  | WA-F1 | 269.3 | 246.1-293.7 | 356 | 332.1-380.4 | -1.50•10^-3^ | 3.07•10^-4^ | -4.89 | **<0.001** |
|  | WA-M1 | 279.3 | 262.6-295.1 | 367.1 | 349.6-386.4 | -1.43•10^-3^ | 2.09•10^-4^ | -6.87 | **<0.001** |
|  | BC-F1 | 2188 | 2049.3-2320.4 | 1899.1 | 1715.1-2089.7 | 3.48•10^-4^ | 1.46•10^-4^ | 2.38 | **0.017** |
|  | BC-F2 | 1922.1 | 1875.7-1969.6 | 1789.5 | 1734.4-1849.5 | 1.56•10^-4^ | 4.48•10^-5^ | 3.48 | **<0.001** |
|  | BC-M1 | 1536.7 | 1488.5-1585.2 | 1568.8 | 1515.5-1622.3 | -3.46•10^-5^ | 3.99•10^-5^ | -0.87 | 0.386 |
|  | BC-M2 (winter) | 1492.6 | 1443.7-1543.9 | 1701.9 | 1643.2-1764 | -2.18•10^-4^ | 4.23•10^-5^ | -5.14 | **<0.001** |
|  | BC-M2 (summer) | 1381.2 | 1343-1421 | 1478.8 | 1430.4-1529.8 | -1.08•10^-4^ | 3.58•10^-5^ | -3.03 | **0.002** |
| *Distance to meadow* | |  |  |  |  |  |  |  |  |
|  | WA-F1 | 792.6 | 749.4-836.1 | 774.1 | 729.9-818.9 | 9.54•10^-5^ | 1.62•10^-4^ | 0.59 | 0.556 |
|  | WA-M1 | 755.5 | 727.7-783 | 638.9 | 609.5-670.5 | 6.53•10^-4^ | 1.20•10^-4^ | 5.44 | **<0.001** |
|  | BC-F1 | 2032.6 | 1854.3-2203.4 | 1697.5 | 1461.2-1934.9 | 2.53•10^-4^ | 1.15•10^-4^ | 2.2 | **0.028** |
|  | BC-F2 | 2140 | 2092.9-2189.2 | 2207.8 | 2134.5-2280.5 | -5.79•10^-5^ | 3.81•10^-5^ | -1.52 | 0.128 |
|  | BC-M1 | 1398 | 1342-1454.1 | 1802.7 | 1727-1878.5 | -2.66•10^-4^ | 3.18•10^-5^ | -8.35 | **<0.001** |
|  | BC-M2 (winter) | 1335.9 | 1279.5-1396.9 | 1673.8 | 1602-1753 | -2.41•10^-4^ | 3.53•10^-5^ | -6.83 | **<0.001** |
|  | BC-M2 (summer) | 1165.2 | 1117.1-1214.3 | 1412.7 | 1350.2-1471.5 | -1.83•10^-4^ | 2.94•10^-5^ | -6.21 | **<0.001** |
| *Distance to road* | |  |  |  |  |  |  |  |  |
|  | WA-F1 | 1014.9 | 951.9-1080.7 | 786.1 | 728.5-848.4 | 5.78•10^-4^ | 1.17•10^-4^ | 4.95 | **<0.001** |
|  | WA-M1 | 1227.5 | 1164.7-1292.5 | 1127 | 1061.8-1193.6 | 1.15•10^-4^ | 5.35•10^-5^ | 2.14 | **0.032** |
|  | BC-F1 | 317.3 | 291.8-341.4 | 208.4 | 181-238.6 | 4.86•10^-3^ | 9.59•10^-4^ | 5.07 | **<0.001** |
|  | BC-F2 | 210.1 | 201.5-218.4 | 206.5 | 197.8-215 | 1.60•10^-4^ | 2.76•10^-4^ | 0.58 | 0.562 |
|  | BC-M1 | 275 | 266.5-284 | 223.5 | 214.7-231.9 | 1.96•10^-3^ | 2.42•10^-4^ | 8.13 | **<0.001** |
|  | BC-M2 (winter) | 274.3 | 265.4-283.3 | 208.4 | 200.5-216.8 | 2.90•10^-3^ | 2.84•10^-4^ | 10.21 | **<0.001** |
|  | BC-M2 (summer) | 279.8 | 272.4-287.1 | 199.9 | 193.1-207.1 | 3.43•10^-3^ | 2.34•10^-4^ | 14.65 | **<0.001** |

**Table S4.** Continued.

| Marten ID | | Used | | Available | | Estimate | SE | Z | P |
| --- | --- | --- | --- | --- | --- | --- | --- | --- | --- |
|  |  | $\bar{x}$ | 95% CI | $\bar{x}$ | 95% CI |  |  |  |  |
| *Distance to salvage-block* | |  |  |  |  |  |  |  |  |
|  | BC-F1 | 520 | 470.2-581.5 | 371.2 | 310.4-440.4 | 1.36•10^-3^ | 4.19•10^-4^ | 3.25 | **0.001** |
|  | BC-F2 | 309.1 | 293.9-324.7 | 191.3 | 176.3-208.2 | 1.63•10^-3^ | 1.63•10^-4^ | 10.02 | **<0.001** |
|  | BC-M1 | 448.8 | 432.1-466.5 | 384.9 | 360.8-411.1 | 3.89•10^-4^ | 9.58•10^-5^ | 4.07 | **<0.001** |
|  | BC-M2 (winter) | 305.1 | 291.9-318.6 | 278.7 | 261.4-296.5 | 3.55•10^-4^ | 1.51•10^-4^ | 2.35 | **0.019** |
|  | BC-M2 (summer) | 343.9 | 331.6-357.3 | 466.5 | 438.9-497.4 | -5.74•10^-4^ | 7.81•10^-5^ | -7.34 | **<0.001** |

**
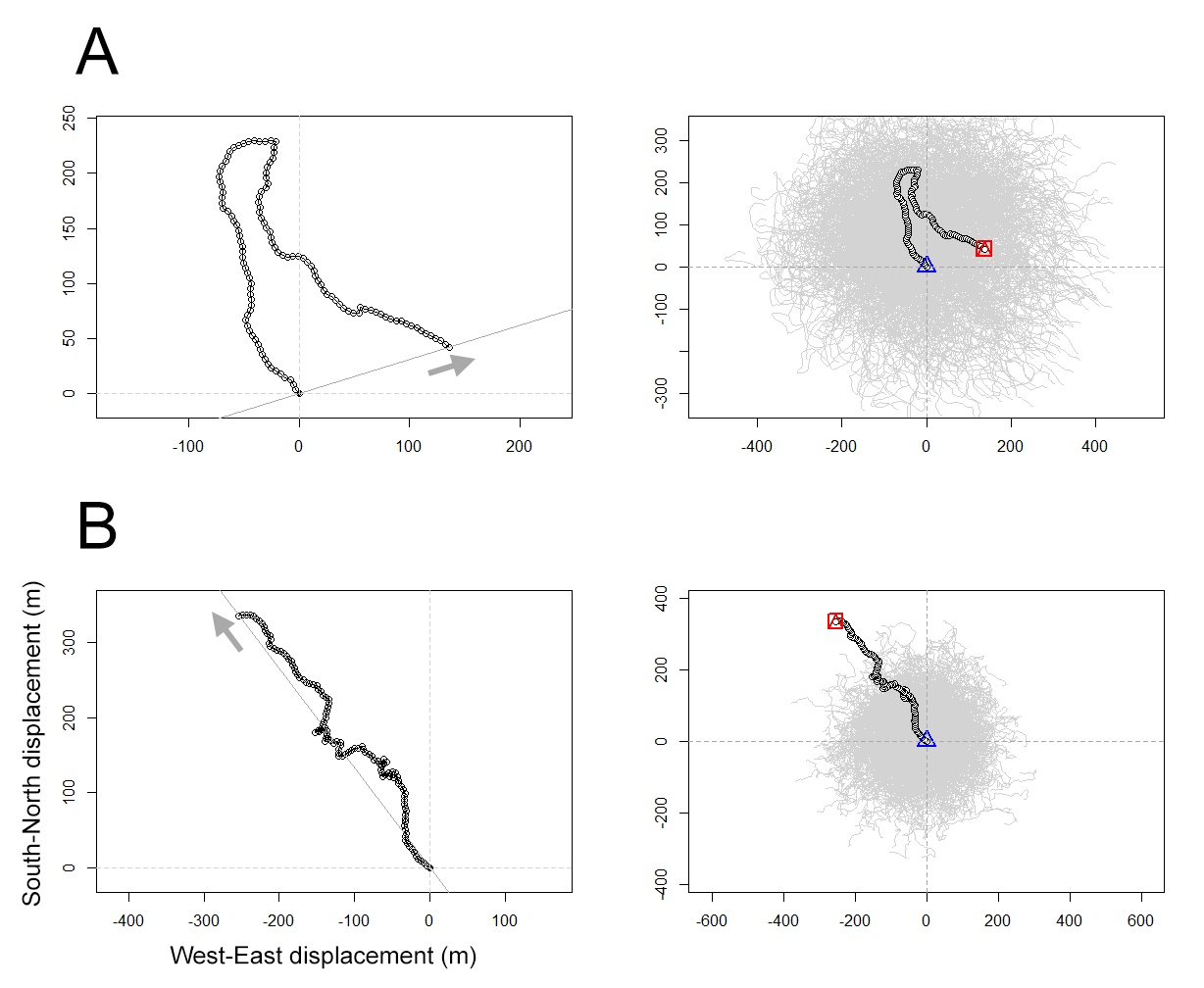
**

**Figure S1.** Marten snow trails range from highly convoluted to essentially straight, and this variation in movement behaviour relates to habitat quality. Row A) shows a random walk characteristic of marten searching high-quality habitat. Row B) is a directed walk expected from marten crossing low-quality habitat. Data are from north-central Washington (2006 burn) in the winter of 2016-2017. Both paths are 600 m long and consist of 120 5 m steps. Arrows indicate the mean direction of movement. Gray areas on the right show 1,000 random walks generated from the same step length, total distance, and turning angle distribution as the original trail. Trails with high “directedness” differ significantly from this random distribution based on Monte Carlo permutation tests.


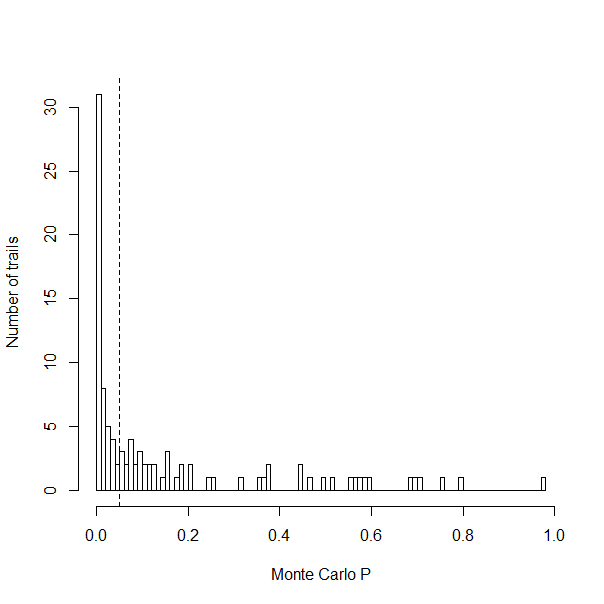


**Figure S2.** Distribution of marten trails in our dataset along a continuum of trail “directedness”, where trails closer to Monte Carlo P = 0 differ more strongly from the null hypothesis of random movement. We considered trails with Monte Carlo P < 0.05, to the left of the dashed line, to represent directed walks; trails with P > 0.05 are more likely to be random walks. Data are pooled across all burns and years.


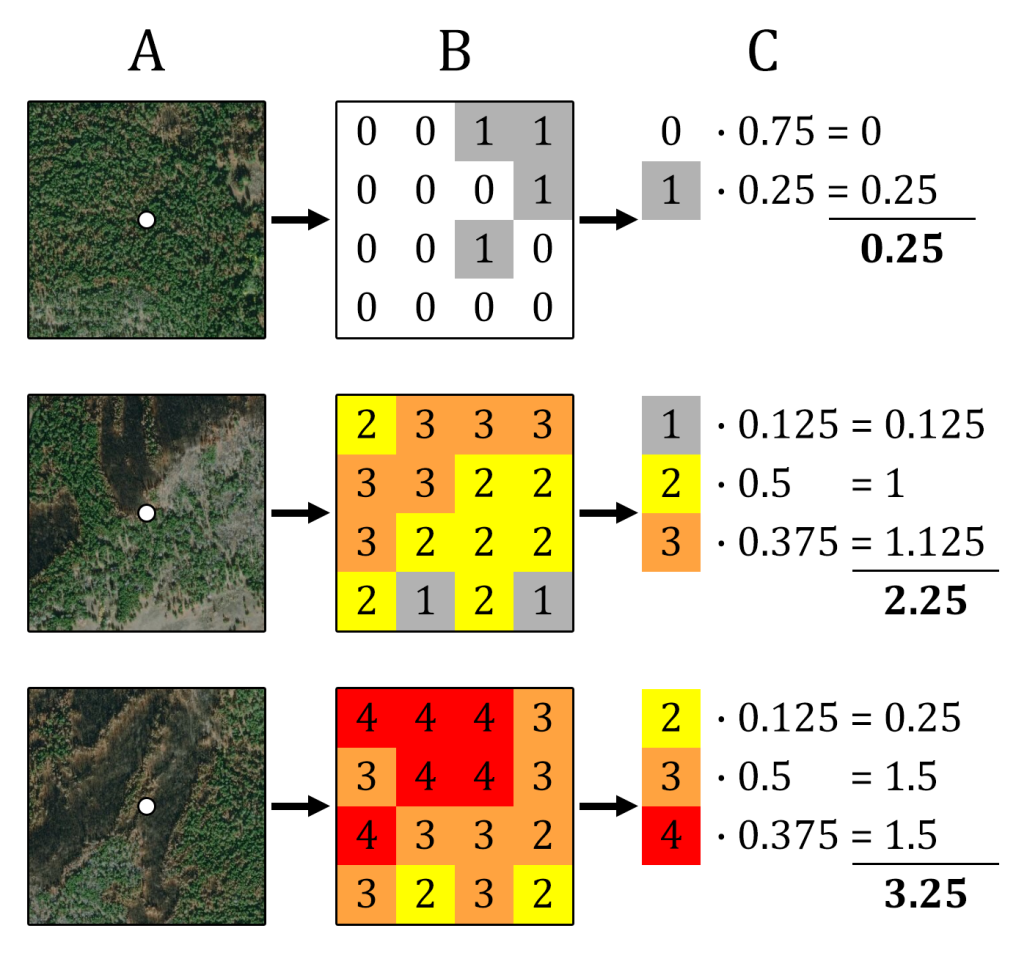


**Figure S3.** “Burn index” measures the overall post-fire burn severity on a portion of the landscape (Roberts et al. 2008). Buffered areas (A) are composed of raster values (B) that characterize burn severity from 0 (unburned) to 4 (high severity). To calculate burn index, these raster values are multiplied by the proportion of each severity class within the buffer—in this simplified example, the number of cells divided by 16. The index thus ranges from 0 if the buffer was entirely unburned to 4 if it burned entirely at high severity; intermediate values represent moderate or mixed severity.


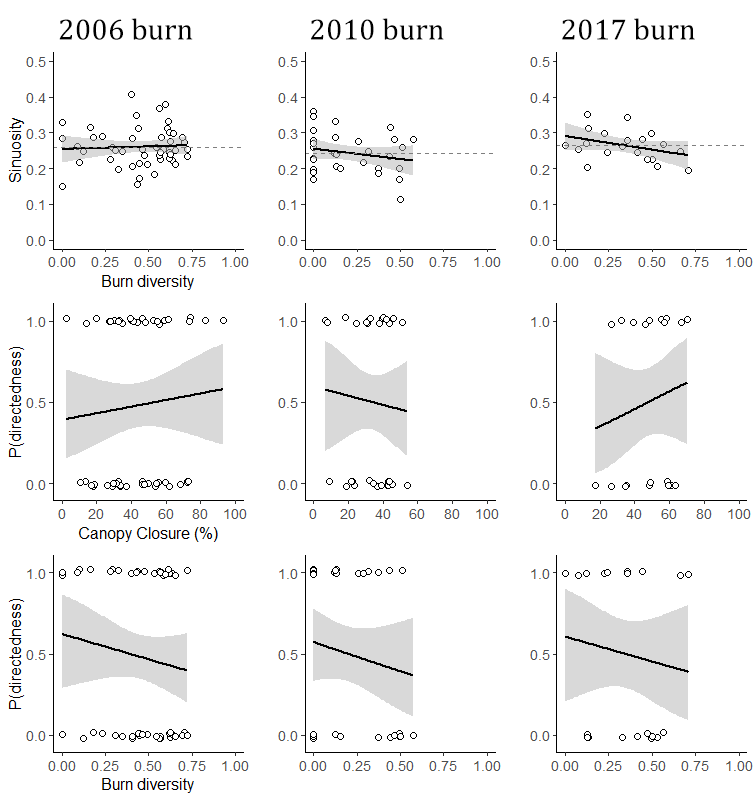


**Figure S4.** Marten movements in response to post-fire canopy closure and landscape heterogeneity in north-central Washington, USA (2006 burn), and central British Columbia, Canada (2010 and 2017 burns), based on linear and logistic regressions. “Burn diversity” measures landscape heterogeneity along a marten trail from 0 (one habitat class dominates) to 1 (all habitat classes in equal proportion). Trails with high directedness, at y = 1, differ significantly from the characteristics of a correlated random walk, and would be expected from marten moving through low-quality habitat. Dashed lines show sample means. All relationships are non-significant. X-axis labels apply to all columns.

**
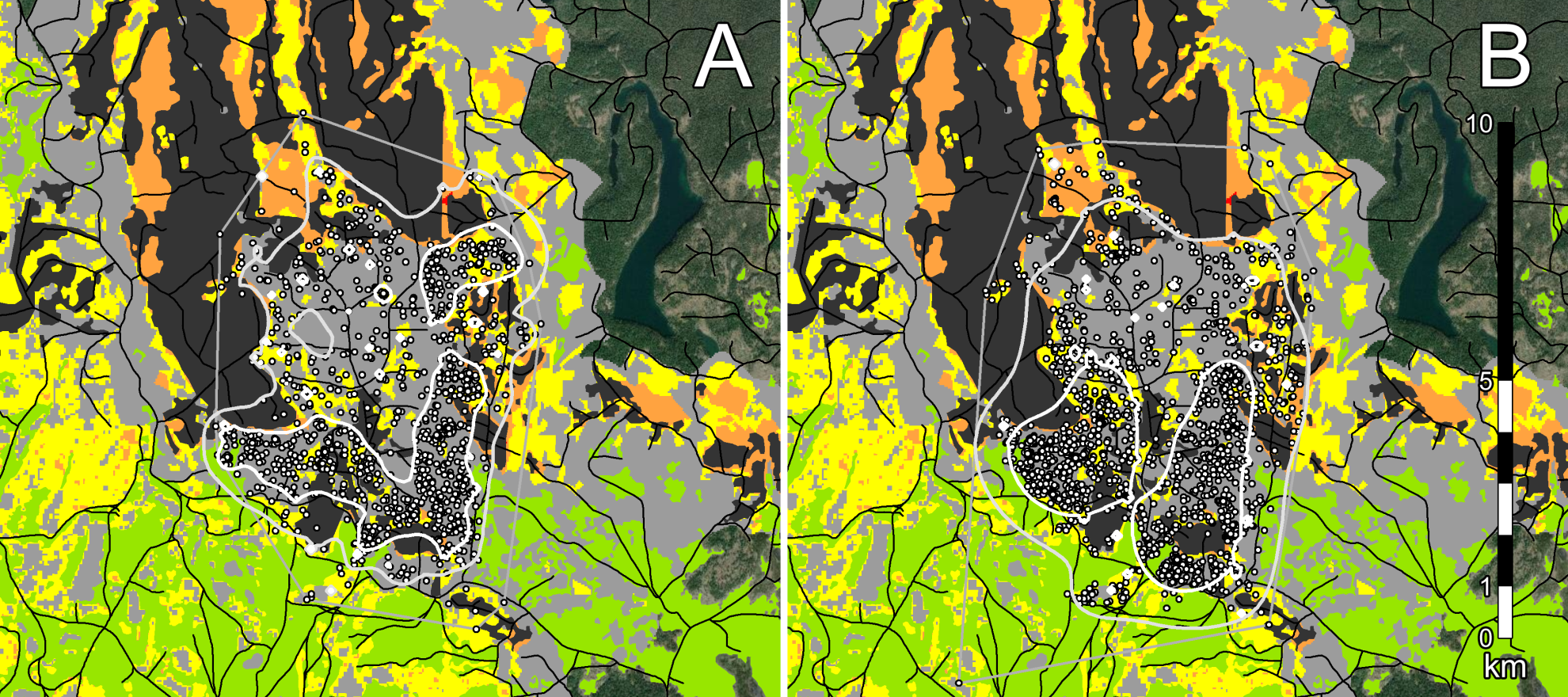
**

**Figure S5.** Post-fire home range fidelity of male marten BC-M2 in central British Columbia, Canada (2010 burn): A) December 2017-March 2018, and B) March-October 2018. Solid lines denote 90% kernel home ranges (light gray) and 50% kernel core activity areas (white); thin gray lines denote convex hulls used in my analyses of habitat selection. Map colours are meadows (green), post-fire salvage-logged areas (darkest gray), and burn severities: gray = unchanged (surface fires), yellow = low, orange = moderate, and red = high. Roads are shown in black.
